# Report on the Progress of Practical Medicine in the Departments of Midwifery and the Diseases of Women and Children, during the Years 1842-3

**Published:** 1844-04

**Authors:** Charles West

**Affiliations:** Member of the Royal College of Physicians, Physician to the Royal Infirmary for Children, and Physician-accoucheur to the Finsbury Dispensary


### REPORT ON THE PROGRESS OF PRACTICAL MEDICINE

IN THE DEPARTMENTS OF

### MIDWIFERY AND THE DISEASES OF WOMEN AND CHILDREN

*During the Years* 1842-3.

By Charles West, M.D.,

**Member of the Royal College of Physicians, Physician to the Royal Infirmary for Children, and Physician-Accoucheur to the Finsbury Dispensary.**

The period included in this Report extends from the first day of October, 1842, to the last of December, 1843. The Report does not profess to furnish a complete index to all that has been written on the general subjects of which it treats during that period; but only to exhibit such facts as are either new, or as furnish new and important illustrations of what was already known, or as serve to confirm the propriety of old modes of practice. It affords no space for elaborate criticism; hence some works, which though of great merit, are occupied rather with the clearer exposition of well-known facts than with the adding to their number, are necessarily passed over without mention. The character of this Report being almost entirely practical, the reader is referred to Mr. Paget's Report in the January number of this Journal, for information on points of Anatomy and Physiology.

The subjects treated of naturally fall under the three heads—Midwifery—The Diseases of Women—and The Diseases of Children,—the minuter subdivisions adopted will, it is hoped, be found convenient.

### I. ON THE PROGRESS OF MIDWIFERY.

#### PREGNANCY.

*Signs of pregnancy.* The changes in the condition of the os and cervix uteri during pregnancy have been investigated by MM. Filugelli,* Chailly,† and Cazeaux.‡ The results they have arrived at agree on the whole with those of Birnbaum.§ M. Filugelli, indeed, appears to have fallen into the error of imagining that the cervix uteri becomes actually elongated in the course of pregnancy; and M. Chailly's paper is principally occupied with the refutation of this opinion; the slight enlargement which may possibly result from tumefaction of the cervix at an early period of pregnancy being in his opinion too slight to be appreciated. M. Cazeaux's conclusions are: 1. That a softening of the texture of the cervix uteri takes place from the very beginning of pregnancy, being for the first few months confined to its lower part, but extending from below upwards, and taking place less rapidly and in a less marked degree in primiparæ than in those who have already borne children. 2. While this softening goes on, the cervix dilates; presenting in those who have had children the form of a funnel with its base downwards, while in primiparæ it is more spindle-shaped. 3. The os uteri is closed in primiparæ until the end of pregnancy; in women who have borne children it is widely open, forming the base of

* Revue Médicale, Nov. 1842. † Annales de la Chirurgie, Jan. 1843. ‡ Ib., Mai 1843.
§ See a notice of Birnbaum's work on the Changes of the Cervix Uteri in Pregnancy in No. XXXI of this Journal.

the funnel. 4. As a general rule, no real shortening of the cervix takes place until about the last fortnight of utero-gestation.

*Disorders of pregnancy.* M. Chailly,* in a paper on *watery discharge from the vagina during pregnancy*, adopts the opinion of Naegele, that the fluid is secreted by the uterus, and not in any way derived from the ovum. The fluid, as it is accumulated, detaches the membranes from the walls of the uterus, and a pouch is thus formed in which it is contained until reaction of the uterus is excited. The contractions of the uterus, however, detach the membranes partially from the cervix; and the fluid escapes in gushes through this aperture, an occurrence that is sometimes painless, sometimes attended with pain about the loins and pelvis. Several cases are related to prove that the uterus, and not the ovum, is the source of this fluid. Thus, a woman in the eighth month of pregnancy suddenly discharged two quarts of reddish fluid from the vagina; and though no sign whatever existed of commencing labour, a similar discharge continued for six days. At the full period labour came on, and it was found necessary to rupture the membranes artificially, when a large quantity of perfectly limpid liquor amnii escaped.

Dr. Cortilhes† calls attention to the frequent complication of pregnancy with *ulceration of the cervix uteri.* These ulcerations are frequently caused by the previous occurrence of abortion, and in many instances exist before the commencement of pregnancy. The symptoms to which they give rise, however, may be so slight as not to attract notice while the patient is unimpregnated, though on the occurrence of pregnancy they generally become very marked; while utero-gestation renders the ulcers very indisposed to heal, or even prevents their cicatrization. The ulcerations are always associated with engorgement of the cervix uteri, which is more considerable in the early months of pregnancy, than at a later period, and they invariably give rise to pain in the lower part of the abdomen. They are usually of an irregularly circular form, four or five lines in diameter, and one or two deep, and have a fungous surface covered with dark red, almost violet-coloured granulations. The ulceration usually begins around the edge of the os uteri and thence extends, giving rise to a thick, yellowish white discharge. Should the disease occur in the earlier months of pregnancy, the ulcerations will often advance, though very slowly, towards cicatrization, and utero-gestation continues undisturbed. When the ulceration supervenes at a later period, no attempt is made at cicatrization, and premature labour comes on unless the woman be subjected to proper treatment. This tendency of the disease to induce premature labour constitutes its gravity; but M. Cortilhes regards it as very amenable to treatment which consists in the local employment of caustics and the use of astringent injections.

Two cases of fatal *hemorrhage* at the end of pregnancy *from the bursting of a varix*, in one instance in the left labium, in the other in the left thigh, are recorded by Dr. Hesse and Dr. Hiller.‡

*Extra-uterine pregnancy.* Of this, many cases have been recorded; they do not, however, illustrate any new point, while the anatomical details of almost all are extremely imperfect. It may nevertheless be useful to refer to them. A case of *interstitial* pregnancy, in which the ovum was situated near the orifice of the left fallopian tube, and which terminated fatally about the third or fourth month, is related by M. Payan.§ Cases of *abdominal* pregnancy are related by Löscher, Hirtz, Nicolai, Lorinser, Wilson, Pohl, and Hauck.|| The patient in Dr. Löscher's case survived eleven years, during the first six of which she was tolerably free from suffering, but afterwards endured much from extreme tenderness of the abdomen, and from attacks of pain, and the formation of a tumour in the right iliac region. She died quite worn out, when the fœtus was found converted into a hard mass, in which was no trace either of placenta or cord. It lay free in the abdominal cavity, and was connected with the viscera only by a few adhesions. The tumour in the iliac region had been

* Bull. Gén. de Thérapeutique, Dec. 1842. † Clinique des Hôp. des Enfans, Sept. 1843.
‡ Medicinische Zeitung, Nov. 30, 1842. § Bull. de l'Acad. Royale de Médecine, Oct. 1843.
|| Neue Zeitschr. f. Geburtskunde, xiii Band, S. 390. Bull. Gén. de Thérap. Mars 1843. Med. Zeitung, June 7, 1843. Oesterr. med. Wochenschr., Feb. 11, 1843. Lond. and Ed. Monthly Journal, Nov. 1843. Oesterr. med. Wochenschr., June 10, 1843. Casper's Wochenschr., Nov. 18, 1843.

formed by the ovaries, both of which were diseased. The fœtus in Dr. Wilson's case sojourned for thirty-seven years in the mother's abdomen, without causing inconvenience. She gave birth to a living child eighteen months after her spurious labour with the extra-uterine child, and died of dropsy at the age of 75. Dr. Pohl's patient recovered perfectly after the lapse of two years, having discharged the bones of the fœtus partly through an aperture in the vagina; partly through the abdominal integuments. Rose, Watson, Creutzer, and Lindsay * have detailed cases of *fallopian* pregnancy ; and Lehwess and Griscom† of *ovarian* pregnancy.

LABOUR.

*Preternatural labour. From causes depending on the mother.* In a series of papers by Dr. R. Knox, of Edinburgh, are some interesting remarks on the *Pelvis.* ‡ He notices the difference in form between the fœtal pelvis and that of the adult female, and endeavours to show that many malformations of the pelvis are the result of arrested development. The fœtal pelvis is of a quadrilateral rather than an oval or rounded form, and its longest diameter is the antero-posterior; in which respect as well as in its general contour, it presents an approximation to the pelvis of the quadruped. The progress of development impresses on it the peculiarities distinctive of the human being, but the fœtal form persists to a certain extent in the pelvis of the male, and its accidental continuance constitutes one variety of malformation of the female pelvis. Female pelves, in which this malformation exists, are characterized by the sides being too straight, the sacrum too narrow, the transverse diameter of the brim too short, and the antero-posterior diameter too long. This arrest of development, however, may affect one side only, and may exist in various degrees. It may either be very slight, or much exaggerated, and in this case may implicate not the form only, but also the size of the bones; which on one side may be positively smaller than on the opposite side. When this deformity exists in any considerable degree, the *pelvis oblique ovata* of Naegele is produced. In illustration of this opinion, he describes three pelves in which this deformity existed. In one, which was the pelvis of a young person, the sacro-iliac synchondrosis was not ossified, although the deformity of the right side was very marked, and exactly such as Naegele has described. In another pelvis too the anchylosis of the sacro-iliac synchondrosis was quite superficial, and did not extend the whole depth of the joint. From these facts he concludes, that anchylosis of the junction between the sacrum and ilium is by no means necessarily connected with this deformity of the pelvis, though it usually follows as a consequence of the arrest of development. [This, indeed, might have been thought probable before, but until the case described by Dr. Knox, no instance was on record in which the obliquely-deformed pelvis had been found unassociated with anchylosis of the sacro-iliac synchondrosis.] In further confirmation of his theory as to the nature of this deformity, Dr. Knox alludes to a pelvis in the possession of Professor v. d'Outrepont, in which this arrest of development existed on both sides. The resemblance it presents to the early form of the fœtal pelvis is very striking, and scarcely less so is its similarity to the pelvis of some of the mammalia, as for instance, the seal; and its whole form affords a remarkable example of the predominance of the law of general type over that which determines the peculiarities of the species. [Dr. Knox speaks of this pelvis of v. d'Outrepont merely from hearsay, but the very elaborate description of it by Dr. Robert,§ and the drawings by which the description is illustrated fully confirm his opinion. Naegele and, after him, nearly all writers on the subject, agree in regarding this form of pelvis as the result of congenital malformation. They conceive, however, that the anchylosis is congenital as well as the malformation of the bones, and by so doing obscure the question of the mode in which this deformity is produced. Dr. Knox has shown that the two are not consentaneous in their occurrence;

* Guy's Hospital Reports, Oct. 1843, p. 488. Edinburgh Med. and Surg. Journal, Oct. 1843, p. 362. Oest. med. Wochenschr., Sept. 2, 1843. Boston Med. and Surg. Journal, August 9, 1843.

† Casper's Wochenschr., Dec. 10, 1842. New York Journal of Medicine, July 1843.

‡ London Medical Gazette, July 14-21-28, 1843.

§ Beschreibung eines im höchsten Grade querverengten Beckens, etc., von Dr. F. Robert; Carlsruhe 1842, Folio.

and to him is due the credit of having been the first to explain the real nature of this malformation, to show that it is not a mere "lusus naturæ" and to elucidate the laws in obedience to which it is produced.]

Dr. Spengel*, in a dissertation written under the auspices of Professor Naegele, describes two cases, neither of which, however, came under his own observation, in which the *pelvis deformed by osteomalacia* yielded so as to admit the passage of the child during labour.

Dr. Ashwell† has made some valuable practical remarks on *Occlusion and rigidity of the Cervix Uteri* as an impediment to labour, and has related cases illustrative of his views. He dissents from the opinion of those who imagine that in many instances of alleged closure of the os uteri, the practitioner was deceived by an unusual degree of obliquity of the uterus placing its orifice out of reach ; and states that he has never met with any case in which labour was seriously protracted by obliquity of the womb. He advocates the early employment of mechanical means to reopen the occluded os, and thinks that, unless its situation be clearly indicated by some marked depression and thinning in the uterine wall, incision is far preferable to any attempt at forcing a passage by the use of a catheter, the finger, &c. He regards the hazard of extensive laceration of the womb following such incisions as an imaginary danger ; and therefore in cases where though the os uteri is not occluded, both it and the cervix are rigid and undilatable, and continue so after the employment of venesection, and the administration of antimony and opium, he conceives incision to be far preferable to any attempt at artificial dilatation.

*Malposition of the uterus*, as an impediment to labour. Dr. Perfetti‡ attended a woman in labour, who had complete procidentia of the uterus. She had suffered more or less from prolapsus uteri ever since she was 15 years old, and on any great exertion the organ appeared externally. Having become pregnant at the age of 22 she was relieved from her ailment until the 7th month of utero-gestation, when it began to return ; at the beginning of the 8th month the uterus reached more than six fingers' breadth beyond the external parts, and during labour it projected still further. After being four days in labour, Dr. Perfetti visited her, and found the os uteri so hard and undilatable as to require incisions to be made into it He then introduced the forceps into the uterus, and extracted the child. The mother recovered, but the prolapsus of the uterus rendered it necessary for her to wear a pessary. Dr. J. Ledesma,§ of Salamanca, has recorded the history of a woman, aged 42, the mother of six children, who was affected with inguinal hernia on the right side. In the 3d month of her 7th pregnancy the hernial swelling suddenly increased in size ; and continued progressively to enlarge up to the period of labour, utero-gestation being undisturbed by this accident. When labour began, the hernial tumour measured 24 inches in length, and 26 in circumference at its broadest part ; its base reached to the crural arch, and its weight had drawn the right labium considerably downwards. When labour-pains came on, which were attended with a slight discharge of liquor amnii from the vagina, an incision was made into the tumour, and a living female child, 22 inches in length, was extracted. The patient would not submit to the division of the adhesions by which the uterus was confined to its abnormal position, and consequently the hernia was not reduced. In 40 days from the date of the operation the patient was sufficiently well to attend to her household duties. [This case is very similar to that which recently occurred to Dr. Fischer, of Berne, mention of which is found in many of the English journals, except that Dr. Fischer's patient died. References to other similar cases will be found in Busch, Geschlechtsleben des Weibes, iii Band, Seite 647.]

*Rupture of the uterus, and laceration of the vagina and perineum.* The note ||

* Dissertatio sistens dilatationem pelvis ex osteomalacia coarctatæ in partubus observatam ; Heidelb, 1842.

† On the Diseases of Women, Part ii, p. 444.

‡ Bulletino delle Scienze Mediche di Bologno, Aprile 1843.

§ Jornal da Sociedade das Sciencias Medicas di Lisboa, t. x, and Oppenheim's Zeitschr., Dec. 1842.

|| Professor Hayn, Casper's Wochenschr., Dec. 31, 1842. Dr. Plath, Oppenheim's Zeitschr., Feb. 1843. Dr. Malcolm, Lond. and Edinb. Monthly Journal, Oct. 1843. Dr. Giese, Casper's Wochenschr., Oct. 7, 1843. Dr. Bowen, Amer. Journ. of Med. Science, Oct. 1843. Sankey, Med. Gazette, Sept. 8, 1843; case of laceration of the vagina.

contains references to cases of ruptured uterus, which presented the symptoms that usually attend that accident, and which terminated fatally more or less speedily after its occurrence. The fatal case of Dr. Giese was associated with putrescence of the uterus, and Dr. Bowen's patient having twice before undergone the Cæsarean secti n, the uterus gave way in the situation of the cicatrix. Besides these cases, four others are recorded, in which the patient recovered * Dr. Mitchell's patient was 38 years old, and the mother of 6 children. From the 6th month of pregnancy she had suffered very severe pain at the lower part of the abdomen. Labour proceeded favorably for the first 12 hours, when sudden collapse and vomiting occurred. The patient was delivered by the crotchet, and it was then found that a rupture existed at the anterior part of the cervix uteri. Extreme irritability of the stomach, and very intractable diarrhœa were the most prominent symptoms that followed her delivery. Opium was given in large and frequently repeated doses, both by the mouth, and in enemata, and on the 31st day after her delivery she was sufficiently recovered to be removed to her own home. M. Castelly performed gastrotomy on his patient three hours after the rupture of the uterus had occurred, and extracted the placenta, as well as the child, through the wound. Severe metro-peritonitis followed the operation, but the patient ultimately recovered. Six months afterwards she menstruated; and nine months afterwards aborted at the third month. The accident to Dr. Vaulpré's patient appears to have been produced by repeated unsuccessful attempts to deliver with the long forceps. Dr. Van Cauwenberge's patient had undergone the Cæsarean section 14 months before. When in the 7th month of pregnancy labour-pains came on; symptoms of ruptured uterus occurred, and the child passed into the abdominal cavity. The exhausted condition of the woman appeared to forbid all interference, but between the 15th and 20th day after the accident happened the cicatrix of the abdominal integuments gave way, and a putrefied fœtus, with its appendages, was extruded. M. Danyau† recommends that the suture should be applied in cases of laceration of the perineum, immediately on the occurrence of the accident, instead of waiting till the patient has recovered from her labour, when it would be necessary to refresh the edges of the wound before applying the suture. The authority of M. Roux is directly opposed to M. Danyau's plan; but M. Danyau asserts that the degree of tumefaction which follows a rent of the perineum has been exaggerated, whilst the suture tends to diminish it, and if proper attention be paid to the introduction of the catheter, and the frequent use of vaginal injections, neither the lochiæ nor the urine will seriously interfere with the healing of the wound. On the other hand, if the operation be delayed, it becomes almost impossible to bring the edges of the wound into contact. In support of his opinion he relates six cases; in five of which the perineum was torn up to, but not into the sphincter, and the operation was successful; in the sixth the sphincter too was involved, and there was considerable ecchymosis about the edges of the wound. The operation, in this instance, failed, sloughing of the parts having taken place on the fourth day after delivery.

*Preternatural labour. From causes depending on the child.* M. Géry relates‡ the case of a woman, in whom *arm presentation* occurred in nine successive labours; her first child having presented naturally; her second by the feet; and the remaining nine having been cross-births References are given in the note§ to cases of spontaneous evolution, which, do not however, add anything to our knowledge of the process as explained by Dr. Douglas. Dr. Hüter, of Marburg, has written a lengthy paper,‖ in which he recommends turning to be practised without rupturing the membranes, in some cases of arm-presentation. The operation is much the same as

* Dr. Mitchell, Dublin Journal of Med. Science, Jan. 1843. M. Castelly, Bull. de l'Acad. Roy. de Méd. Sept. 30, 1843. Dr. Vaulpré, Gaz. Méd. Mars 18, 1843. Dr. v. Cauwenberge, L'Expérience, Nov. 18, 1843. † Journal de Chirurgie, Juin 1843. ‡ Revue Médicale, Nov. 1842.

§ v. Hammer, Neue Zeitsch. f. Geburtsk. xiii Band, S. 75. Corbyn, India Journal of Med. Science, Dec. 1842, relates two cases. Hinterberger, Oest. med. Wochenschr., March 25, April 1, 8, 1843, relates seven cases, in two of which there were twins, and the evolution took place with the second child. Two children were born alive, but died soon after birth ; one of these was a twin, the other was not, and the mother had reached the full term of utero-gestation. Huguier, Gaz. des Hôp., Août 24, 1843.

‖ Neue Zeitsch. f. Geburtsk., xiv Band, S. 1.

that practised by Michaelis, when prolapsus of the cord occurs, and consists in the introduction of the hand between the uterus and the membranes, until the operator reaches the feet, knee, or other part, when, without rupturing the membranes, it will be extremely easy to turn the child. He practised this manœuvre in five cases, but in three the same person was the subject of the operation. In four of the five cases the child was saved, and in one instance the mother had previously lost two children by the ordinary mode of turning. He recommends this practice as disturbing the ordinary course oflabour much less than the usual mode of turning, since the rupture of the membranes is left to nature, and the case may be afterwards managed, as if the presentation had been natural from the beginning. The whole quantity of the liquor amnii being available in this operation, its performance is greatly simplified, while it has the further advantage of avoiding the prolapse of the cord, or of admitting of its reposition, should that accident have occurred. He denies that the uterus would be much irritated by this proceeding, and asserts that the membranes would be stretched, and forcibly separated from the uterus, or the placenta partially detached, only, if the operation were attempted before the os uteri is sufficiently dilated, or if the membranes were morbidly adherent, while auscultation would always be an adequate guide to the situation of the placenta. [These arguments do not appear by any means conclusive; the various favorable circumstances which, according to Dr. Hüter, warrant the operation, are not found often to coincide, and the hazard of detaching the placenta is probably much greater than he represents it to be. His assertion, too, that auscultation would invariably guide to the situation of the placenta is incorrect, for in 180 out of 600 cases, in which it was resorted to by Naegele,* or in 30 per cent., it was not possible to determine the seat of the placenta.]

A descendant of the famous Saxtorph has published a dissertation† on *Prolapsus of the cord*, which, without containing anything new, is a very valuable summary of the present state of knowledge, and obstetric practice in such cases.

Instances in which labour was impeded by *malformation of the fœtus* are related by M. Bouchacourt,‡ and Dr. Porter.§ In the former case the abdomen of the fœtus was enormously enlarged by hydatids of the kidneys, and delivery could not be accomplished until after its evisceration; in the latter the obstacle arose from an enormous spina bifida occupying the sacrum.

*Preternatural labour. From abnormal condition of the ovum.* A case of *Partus siccus* is detailed by M. Matthysen,‖ in which the patient was delivered of her first child after a quick labour, but in which no discharge of liquor amnii either preceded or followed its birth. The placenta came away naturally, the uterus contracted well, and though the lochiæ were exceedingly scanty, no accident occurred in the puerperal state. The child was born alive, full grown, but extremely thin, and its skin was covered by a thick coating of vernix caseosa. [M. Matthysen erroneously supposes this to be the only case of the kind on record; instead of which many cases have been related since attention was first called to it by Rudolphi.¶]

*Operative midwifery.* MM. Pereira and Lasserre,** formerly *internes* at the Maternité, have published an essay on the abuse of various obstetric manœuvres, the accidents to which they give rise, and the advantages of temporising in the practice of midwifery. Many cases are related, illustrative of the evil consequences of forcible dilatation of the cervix uteri, and of the application of the forceps, with no other indication than the slowness of the labour. In this last category there are related several cases of laceration of the vagina, and rupture of the uterus, and the remarks of the writers on these accidents form the most valuable part of the paper. They next endeavour to show, by the evidence of statistics, that the danger to the mother's life, from the mere prolongation of labour, is less than the danger to which she is exposed when the labour is terminated by manual or instrumental interference.

* On Obstetric Auscultation, West's translation, p. 80.

† De Prolapsu Funiculi Umbilicalis; Havniæ, 1842. See also, for a review of it, Br. and For. Med. Review, April, 1843.

‡ Bull. Gén. de Thérapeutique, tome xxiii, p. 473.

§ American Journal of Med. Science, April 1843.

‖ Annales d'Obstetrique, Janvier 1843.

¶ De Partu Sicco; Erlangæ, 1790.

** Archives Gén. de Med., Janvier et Fevr. 1843.

*Craniotomy.* Dr. Smith,* of Glasgow, has invented a new perforator; the blades of which expand, by means of a screw, much in the same way as the blades are separated in Weiss's speculum. He conceives that by this modification some of the dangers incidental to the employment of the ordinary perforator are avoided. Various attempts have been made to improve Baudelocque's cephalotribe:† many of these devices display much ingenuity, but at the same time add greatly to the complication of this formidable and, it may perhaps be added, useless and dangerous instrument.

*The Cæsarean section.* The note‡ contains references to various cases of this operation [which with those collected by Kayser, make a total of 349, of which 131 terminated favorably for the mother; while in 218, her life was sacrificed.] There are besides, two other cases that occurred within this period, in which the operation was performed, after the death of the mother, and in one case, that of Dr. Loweg, the child's life was preserved.§

*Uterine hemorrhage.* Dr. Lever‖ calls the attention of the profession to the peculiar tendency to uterine hemorrhage induced by disease of the spleen and by granular degeneration of the kidney. He relates three cases of disease of the spleen in which uterine hemorrhage came on immediately after delivery, and mentions having met with others of a similar kind. From these he concludes that in persons affected with disease of the spleen there is a peculiar tendency on the part of the uterus to dilate, and thus to admit of internal hemorrhage. The coagulated blood, too, retained in the uterus gives rise to considerable irritation, marked by rigors, fever, &c.; the fever being very apt to assume the intermittent type, especially in persons who have once suffered from ague, and is cured by the same remedies as other intermittents. Two cases of hemorrhage in persons affected with Bright's kidney are related; puerperal peritonitis subsequently came on, and the patients died. v. D'Outrepont¶ recommends the employment of a strong solution of chloride of iron, as a styptic in cases of uterine hemorrhage. He injects an ounce of a saturated solution of the salt into the vagina, and in a case of placenta presentation the plug which he employed was dipped in this solution.

### THE PUERPERAL STATE.

*Puerperal convulsions.* Dr. Johns,** assistant at the Dublin Lying-in Hospital, calls attention to the fact that puerperal convulsions never occur in labour without premonitory symptoms having existed during pregnancy. The most frequent symptoms are swelling, not limited to the lower extremities, but involving the hands, arms, neck, and face. If besides there be headach, sense of weight or giddiness in the head, ringing in the ears, a temporary loss of vision, or severe pain in the stomach with flushings of the face, the risk of convulsions is considerable. This risk amounts to almost absolute certainty if the woman be pregnant for the first time, or have suffered similarly in former pregnancies, if she be full and plethoric, and if the presentation of the child should be a natural one. He gives full statistical details to show the liability of primiparæ to convulsions, and also to prove the more frequent occurrence of convulsions in cases where the presentation is natural. Dr. Lever†† notices the frequent connexion of an albuminous state of the urine, with puerperal convulsions, and suggests that congestion of the kidney from pressure of the gravid uterus on the renal veins is its probable cause. He grounds his opinion on the circum-

* London and Edinburgh Monthly Journal, Nov. 1842.

† Finizio, Annales d'Obstetr., Nov. 1843. v. Huevel, Ibid., Oct. 1843. Chailly, Bull. Gén. de Thér., Fevr. 1843. Cazeaux, Annales de la Chirurgie, April 1843. Langheinrich, Neue Zeitschr. f. Geburtsk., xv Band, S. 110.

‡ Cases in which both mother and child were saved: Ringens, Med. Zeitung, Nov. 23, 1842 Monin, L'Expérience, 6 Avril, 1843. Schacht, Casper's Wochenschr., May 13, 1843. Berndt, Neue Zeitschr. f. Geburtsk., xiv Band, S. 335. Cases in which the child only was saved: v. D'Outrepont, Neue Zeitschr. f. Geburtsk., xiii Band, S. 431. Dr. C. Falconer, Amer. Journal of Med. Science, July 1843. Cases in which neither mother nor child was saved: Wraith, Prov. Med. Journal, Jan. 21, 1843. Hooper, Lancet, Feb. 4, 1843.

§ Lorinser, Oester. med. Wochenschr., Jan. 1, 1843. Loweg, Casper's Wochenschr., Dec. 2, 1843.

‖ Guy's Hosp. Reports, Oct. 1842, p. 325.

¶ Neue Zeitschr. f. Geburtsk., xiii Band, S. 322.

** Dublin Med. Journal, Sept. 1843.

†† Guy's Hospital Reports, Oct. 1843.

stance that in nine out of ten cases of puerperal convulsions in which the urine was examined, it was found to contain albumen; a condition which is shown not to be generally incidental to parturient women by the fact, that the urine of fifty women in labour was examined without a trace of albumen being discovered, except in one or two who had shown premonitory symptoms of convulsions. To this albuminous state of the urine he is disposed to refer the œdema of the face and extremities, which has been noticed by various writers as premonitory of puerperal convulsions, and indicating the employment of most active antiphlogistic measures. A similar opinion has been expressed by Dr. Simpson,* who states that for the past two years he has been accustomed to teach his class "that patients attacked with puerperal convulsions had almost invariably albuminous urine, and some accompanying or rather preceding dropsical complication, and hence, probably, renal disease;" and in one instance he had the opportunity of confirming this supposition by a post-mortem examination. Somewhat connected with these inquiries is the paper of M. Lasserre† on metastatic serous congestions in women recently delivered He notices the œdema of the extremities which often exists in pregnant women, owing in part to, what he terms, a serous diathesis; but still more to the mechanical obstacle to the return of blood from the lower parts, caused by the pressure of the gravid uterus. After delivery, this obstacle is removed and the œdema disappears, often very rapidly. When the œdema has been very considerable, and its subsequent removal rapid, the sudden introduction of a large quantity of serum into the circulation gives rise to a true serous plethora, which is characterized by a full and hard pulse, and considerable constitutional disturbance. In most cases the absorption of serum does not go on very rapidly until after the secretion of milk has been established: and the latter function seems to counteract any tendency to constitutional disturbance which the former process may have given rise to, or arrests it before it becomes serious. He relates instances to illustrate this; in one of which cerebral symptoms subsided so soon as a copious secretion of milk was established, while at the same time the urine became albuminous and greatly increased in quantity.

Should the œdema disappear very rapidly without milk being abundantly secreted, various accidents may occur, which generally assume a grave character about the third day after delivery, though they appear earlier in a slight degree. The first symptoms are a return of the œdema about the face and eyelids, and a general hebetude of the senses. Respiration is often slightly laborious, the pulse is large, full, and hard, and the cellular tissue of the extremities becomes œdematous. The lochiæ continue abundant, and soon after delivery grow very pale and serous. These symptoms may disappear under judicious treatment, or they may be succeeded by a state of coma growing by degrees more and more profound, and by other symptoms resembling serous apoplexy, and proving very speedily fatal. The sinuses in such cases are found gorged with very fluid blood, and an immense quantity of serum is infiltrated into the sub-arachnoid tissue, and contained in the ventricles; and the pleuræ also often contain a large quantity of fluid. Sometimes the lungs, rather than the brain, become the seat of this congestion; urgent dyspnœa occurs, moist râles are heard through the whole of both lungs, and the patient dies with intense bronchitic symptoms within twenty-four hours from the time of their first assuming an alarming character. After death the lungs are found highly œdematous, the bronchi much congested, and the pleuræ full of fluid. Punctures of the infiltrated extremities as a prophylactic measure, and the most vigorous employment of depletion, and evacuants, as antimony and ipecacuanha, whenever serious symptoms come on, constitute the treatment.

*Puerperal fever, &c.* Reference must be made to the very valuable lectures of Dr. R. Lee,‡ which, however, merely afford a confirmation of opinions expressed in his former work. Nothing, indeed, actually new has appeared on the subject of puerperal fever and uterine inflammation; but Dr. Doherty,§ in a paper on *Chronic*

* London and Edinb. Monthly Journal, Nov. 1843, p. 1015, note.
† Gazette Médicale, Nov. 25 and Dec. 2, 1843.
‡ Published in the Medical Gazette, and since reprinted in one volume.
§ Dublin Journ. of Medical Science, Nov. 1842.

*Inflammation of the Uterine Appendages after Parturition*, calls attention to a disease which has not been noticed so much as its importance deserves. It is not usually announced by any very severe symptoms; but a woman who, to all appearance, had recovered perfectly from an ordinary labour, is seized, after exposure to cold, days or weeks afterwards, with shivering, febrile symptoms, and a dull sense of weight about the pelvis. Febrile paroxysms recur at intervals; as they grow more frequent, pain and difficulty in moving the leg of the affected side are felt. The patient still walks about, her pulse is rapid but soft, rigors are frequent, pain and difficulty in moving one or both legs are experienced, there is frequent desire to pass water, and tendency to diarrhea. Uneasiness is felt in one or both iliac fossæ where there are fulness and hardness, and tenderness on pressure, especially in the course of Poupart's ligament. Further indications of the disease are discovered on making an internal examination, when the vagina will be found tender, firm, and inelastic, and the uterus bound down more or less completely to the affected side. When the disease has advanced further, the ovary may often be felt externally as a round circumscribed tumour, but it is by examination per rectum that the ovarian tumour will be most certainly distinguished; [a point, by the bye, first insisted on by Loewenhardt, in his remarks on inflammation of the ovary. See Br. and For. Med. Rev., vol. 11, p. 527.] A paper by Dr. Churchill* treats of the more chronic form of this affection, and forms a kind of sequel to Dr. Doherty's essay. He illustrates his remarks by the detail of numerous cases, partly original, partly recorded by other writers. He calls attention to the fact, that though considerable local pain, as well as general febrile symptoms are usually present, yet the disease may proceed unattended by either, and the existence of a tumour in the iliac region may be the first sign of its occurrence.

*Lactation. Its influence on conception.* Dr. Loudon,† in a work on the law of population and subsistence, propounds the theory that the laws of nature require lactation to be prolonged for three years, and expresses an opinion that the antagonism between the uterus and mammæ is so great as usually to prevent conception in women who have infants at the breast. This opinion, however, does not accord with the facts stated by Mr. Roberton, and is even more decidedly at variance with the results arrived at by Dr. Laycock. Dr. Laycock‡ states that 135 married women yielded 209 pregnancies during 766 lactations, or 1 pregnancy in 3·66 lactations, or 27 per cent. These 209 pregnancies occurred in 76 females, that is to say 56 per cent. became pregnant while suckling; but in 30 of these pregnancy under these circumstances occurred only once. If, therefore, they be deducted, there remain 46, or 33·9 per cent., or nearly 1 in 3 who became pregnant on more than one occasion while suckling; and 19 of these, or 1 in 7, had always (after their first pregnancy) conceived while suckling.

*Influence of menstruation on lactation.* M. Raciborski§ investigated the influence of menstruation on the milk of nurses, and on the health of the infant in seven women who menstruated while suckling. He could discover no other change in the milk at those periods, than that it contained a smaller quantity of cream; and he concludes that the injurious influence of menstruation on the health of the infant has been greatly exaggerated, and that the circumstance of a woman menstruating during lactation is not a sufficient reason for rejecting her as a wet nurse.

*Diseases attending Lactation.* Dr. Shanks‖ of Memphis in Tennessee, and Dr. Taylor¶ of Monticello in Florida, describe a peculiar form of sore mouth, incidental to suckling women, and which is apparently owing to endemic causes. It comes on in the latter months of pregnancy, alternating with diarrhea, and being attended with great derangement of the digestive powers. It usually ceases for a short time after parturition, but soon returns with increased severity, associated with diarrhea, which sometimes wears out the patient, and thus proves fatal. When the disorder begins, the tongue and whole interior of the mouth become red and raw, and a burning acrid

* Dublin Journal of Medical Science, Sept. 1843.

† Solution du Problème de la Population, etc.; Paris, 1842.

‡ Dublin Med. Press, Oct. 26, 1842.

§ Bull. de l'Acad. Roy. de Méd., Juin 15, 1843.

‖ American Journal of Med. Science, Oct. 1842. ¶ Ibid., Jan. 1843.

saliva is secreted. A state of feverish excitement accompanies this condition in plethoric females, while those of a weaker frame are free from fever. This state alternates with diarrhea, but as has already been mentioned, they both cease for a few weeks after parturition, but then usually return. The diarrhea is almost or altogether painless; the evacuations are copious, thin, and dirt-coloured; or in the more protracted forms ash-coloured; or light, fermented, and frothy. The soreness of the mouth is most distressing, being sometimes so extreme, that any attempt to speak, or to swallow the blandest food produces pain. The secretion of milk continues copious, and the infant thrives for a long time after the disease has become very severe The gradual loss of strength from the continued diarrhea and the general nervous irritation give rise to a fatal issue in the worst cases. If the disease should occur during pregnancy, a generally alterative plan must be adopted, and, sometimes in plethoric persons, depletion must be had recourse to, and a strictly antiphlogistic treatment be pursued. During lactation alteratives must be employed, with a very strict diet; ipecacuanha with or without opium is useful; active purgatives are always bad; but in obstinate cases small doses of arsenic or corrosive sublimate may be employed; and weaning the child sometimes becomes absolutely necessary to the mother's recovery. Dr. Shanks has found an infusion of the sanguinaria canadensis form the best local application for the mouth. He attributes the disease to marsh miasmata, since it never occurs in high and dry districts, and has diminished at Memphis, in proportion as the country has been cleared, and endemic fevers have become less frequent. A form of diarrhea with a somewhat similar affection of the mouth is not unusual among males in the same district; and on this fact, as well as on the nature of the remedies which he has found beneficial, he grounds his opinion as to its cause. Dr. Taylor, however, is inclined to dispute the influence of local causes in its production, since he has met with it in very different situations.

### II. ON THE PROGRESS OF KNOWLEDGE WITH REFERENCE TO THE DISEASES OF WOMEN.

### DISORDERS OF MENSTRUATION.

*Amenorrhea.* M. C. Bécasseau* relates a case of amenorrhea, dependent upon *imperforate cervix uteri.* The subject of the observation was a young woman, aged twenty-six, whose health had been good until her twenty-third year; when menstrual molimina came on, attended with hysteria, epilepsy, and hemoptysis, recurring every month. A membrane was found occupying the fundus of the vagina, and completely concealing the cervix uteri. After its division and the exposure of the cervix no trace of the os uteri could be seen beyond a slight depression, but a puncture having been made in that situation, gas escaped mixed with fetid blood, and menstruation afterwards occurred regularly at every period. M. Böhm† details two cases, in which, during convalescence from typhus fever, inflammation of the vagina occurred, followed by purulent discharge, and the separation of the lining membrane of the vagina in a sphacelated state. This was followed by adhesion of the walls of the vagina, and consequent retention of the menses. Operative means were had recourse to: in one case with perfect success; in the other a putrid fever came on five days afterwards, and destroyed the patient.

*Menorrhagia.* Dr. Stevenson‡ and Dr. Simpson, § both recommend the gallic acid as a very valuable astringent. Dr. Stevenson gave it in doses of eight grains every three hours; and in three very alarming cases which he relates, the action of the remedy was speedy and satisfactory. Dr. Simpson has found it very useful, though not invariably so, in many extremely obstinate cases. He gave it during the continuance as well as in the intervals of the discharge, in doses of ten to twenty grains in the form of pills, daily; and finds that it has the great advantage over many other antihemorrhagic medicines, that it does not constipate the bowels.

* Gazette des Hôpitaux, Oct. 15, 1842. † Oesterr. med. Wochenschr., Feb. 25, 1843.
‡ Edinburgh Med. and Surgical Journal, July 1843, p. 103.
§ London and Edinburgh Monthly Journal, July 1843, p. 660.

### DISEASES OF THE VAGINA.

*Vesico-vaginal fistula.* M. Lallemand* has published the paper on this subject which was read at the Institute, in August, 1839. He observes, that vesico-vaginal fistula, from any other cause than difficult labour, is of extremely rare occurrence; that even carcinomatous ulceration very seldom gives rise to it. The transverse form which the aperture almost invariably assumes is partly owing to the form of the pubic arch against which the fœtal head presses; but is likewise in part produced by the transverse direction of the folds of the vagina favouring the diminution of the long diameter of the fissure. The proximity of the fissure to the neck of the bladder, or its remoteness from it, has been supposed to modify the degree of discomfort experienced by the patient; but M. Lallemand denies that in this respect there exists any real difference, except between *urethro-* and *vesico-vaginal fistulæ.* The power of retaining much or little urine depends on the size of the fistula, rather than on its situation.

Any attempt at the cure of a fistula is justifiable only in cases where it is not so large but that its edges can, for the most part, be brought into contact; and eighteen lines in its longest diameter is the largest size of any fistula on which M. Lallemand has ever operated with success. In all cases, however, the state of the patient's general health has a great influence on the healing process.

Fistulæ so small as to be closed by the tumefaction of their edges may be healed by cauterization alone, provided a catheter be kept in the bladder; and the same result may often be attained with larger fistulæ by a frequent repetition of the operation. In cases where the cure of fistulæ is attempted by paring their edges and then employing the suture, the distance of the fistula from the entrance of the vagina greatly increases the difficulties of the operation; no such result, however, occurs when the actual cautery or lunar caustic is employed, and the edges of the wound are kept in contact by the *sonde-airigne.* It should, however, be borne in mind, that all operative proceedings are hazardous in the case of fistulæ which are close to the cervix uteri. Fatal peritonitis has been induced in such cases; probably owing to the proximity of the peritoneum at that part where it passes from the uterus to the bladder.

The age of the fistulæ has a great influence on their curability: the older they are, the more difficult are they of cure; and, in no case that terminated successfully, had more than a year elapsed from the time of their occurrence. This, however, does not depend on any increased hardness of the edges of the wound, but results from the deposit on them of the salts of the urine, and from the various circumstances connected therewith, which impair the patient's health. This deposit of the salts of the urine, too, is not only unfavorable in itself, but also inasmuch as it betokens that the irritation has extended from the bladder to the kidneys; and, consequently, that the general condition of the constitution is unfavorable to the reparative process.

M. Lallemand is opposed to the practice of paring the edges of the fistula, and then attempting their reunion by the suture, since the size of the opening is thereby increased without any of that swelling being induced which follows cauterization, and often with so beneficial a result. It is his custom to employ cauterizing irons, the stem of which is made as slender as possible, except in cases where the fistula is very minute and sinuous, when he uses the lunar caustic; since irons, sufficiently delicate for the purpose, would not retain their heat. As a means of bringing the edges of the wound into contact after cauterization, he prefers the *sonde-airigne* to any other instrument. This is a female catheter, of large caliber, from the bottom of which hooks are made to project, by means of a spring, after it has been introduced into the bladder. These hooks pierce the substance of the vesico-vaginal wall, and bring the edges of the wound into apposition. The cicatrix ought not to be examined for a fortnight. He has employed the suture twice, and, on both occasions unsuccessfully; but he has employed the *sonde-airigne*, after cauterization, fifteen times, and in nine of these with success. All of these nine cases were cured by the second, many of them by the first operation.

Dr. James Reid† recommends, as a valuable palliative in cases apparently incura-

* Archives Gén. de Médecine, Mars 1843. † Lancet, Feb. 18, 1843.

ble, the introduction into the vagina of a caoutchouc bottle, to which a female screw and stopcock have been attached, and then distending it with air, by means of a syringe, until the patient cannot conveniently bear its further distension. The bottle may be withdrawn, for the purpose of injecting the vagina, &c., by merely letting out the air. Two cases are recorded, in one of which the catheter also was used, though not regularly, in the other it was not employed at all; and both patients were treated under great disadvantages. The use of the indian rubber bottle, however, was followed by complete closure of the fistula in one of these cases, while in the other the patient was placed by it in a state of comfort, and enabled to retain her urine for a considerable time. [This plan seems preferable to the mode recommended by M. Leroy d'Etiolles,* who cuts a leaf of caoutchouc, three or four fingers' breadth long, and having applied it to the fistula, plugs the vagina with balls of charpie rolled in caoutchouc reduced to a kind of paste. With these balls of charpie thus invested with indian-rubber, he can distend the vagina more or less, according to circumstances. The ordinary caoutchouc bottles hitherto employed in these cases have this disadvantage, that it is not possible to regulate the distending power they exert.]

### DISEASES OF THE UTERUS.

Dr. Lever's† valuable work contains some interesting observations on the *frequency of organic diseases* of the uterus. He concludes, from the investigation of 2588 cases, that 31 per cent. of all diseases of the uterus are organic; or, including also the data furnished by others, that 34·7 per cent. are organic, and 65·2 per cent. functional.

Dr. Simpson‡ animadverts, in a series of papers, on the insufficiency of any of the present means of investigation for forming a correct *diagnosis* in many varieties of *uterine disease*. To supply this deficiency he recommends the employment of an *uterine sound* or *bougie*, by which when introduced into the uterine cavity it is possible "to ascertain the exact position and direction of the body and fundus of the organ; to bring these higher parts of the uterus in most instances within the reach of tactile examination, and to ascertain various important circumstances regarding the os, cavity, lining membrane, and walls of the viscus." This instrument resembles a common male sound, but is rather smaller; it is bulbed at the end to prevent its injuring the uterus, but tapers gradually from the handle to the bulb, being one fifth of an inch in diameter at the former, one tenth of an inch at the latter situation; and the bulb is one eighth of an inch in diameter. Its stem is nine inches long, and it is graduated in order to measure the dimensions of the uterus. There is a small knob on the stem two inches and a half from the bulb, that being the ordinary length of the uterus, and the further graduation of the instrument is effected by a series of shallow double grooves, half an inch or an inch from each other.

The introduction of the instrument is said to be usually attended with but very slight uneasiness, and in a few cases only with feeling of sickness; the occurrence of any actual pain indicates that the lining membrane of the uterus is not in a healthy state. Dr. Simpson proposes applying this instrument to ascertain many obscure points in cases of uterine disease; his recommendation of it, however, appears to be often founded on reasoning, rather than on actual trial. He proposes its employment to rectify various malpositions of the uterus [in which he appears to have been partially anticipated by Osiander,§ who employed his dilatorium orificii uteri with success to effect the reposition of a retroverted uterus.]

*Displacements of the uterus. Prolapsus uteri.* Mr. Snow|| has suggested a useful modification of the sponge pessary, which consists in enveloping a piece of sponge, of the proper size and shape, in oiled silk, and tying it firmly at one end, so as to leave a small stem or tail, half an inch in length, by which it can be removed. An instrument-maker of Paris, M. Bienaimé¶ has invented an apparatus for prolapsus

* Gazette des Hôpitaux, Sept. 13, 1842.
† Practical Treatise on the Organic Diseases of the Uterus; London, 1843.
‡ London and Edinburgh Monthly Journal, June, August, and November, 1843.
§ Kilian, Operationslehre f. Geburtshelfer, ii Theil, S. 211.
|| Med. Gazette, April 14, 1843.
¶ A description and engraving are given in Annales d'Obstétrique, Août 1843.

uteri very similar to Hull's bandage. Dr. Simpson* proposes, and has used with success in an instance of very aggravated procidentia, a modification of the ordinary pessary. This consists, in the addition to the common stem and cup-shaped pessaries, of a short central stalk of nickle or silver wire, which is passed into the cavity of the cervix, so as to maintain the replaced organ in the cup of the instrument.

*Organic diseases of the uterus. Hypertrophy of the uterus.* Dr. Arnal† recommends the aqueous extract of the ergot of rye in hypertrophy and other chronic affections of the uterus. Active inflammation contra-indicates its use, but in chronic engorgements of the uterus, in uterine catarrh, leucorrhea, and those œdematous engorgements which accompany the chlorotic habit, it was uniformly beneficial. The aqueous extract has from five to six times the strength of the powder; it was given by M. Arnal at first in small doses, which he gradually increased until the patients took about fifteen grains daily, and this treatment was continued on the average for three months. No disturbance of the circulation followed its employment, nor was any effect produced on the general health except that slight pains were experienced in the uterine region, similar to those which precede menstruation. The other remedies employed were repose, the use of the hip-bath, and emollient or slightly astringent injections into the vagina; and in cases of chlorosis or chronic leucorrhea, the iodide of iron was advantageously combined with the ergot. This treatment was employed with uniform success in 36 cases; ulceration of the cervix uteri coexisted with the other affection in 22 instances; but as a general rule, the cure of these ulcers was not found to be expedited by cauterization.

M. Jobert‡ describes a peculiar affection of the unimpregnated uterus, which may be confounded with hypertrophy of the cervix. It consists in the distension of the cervix by fluid, which excites uneasy sensations, but escapes, with considerable relief to the patient, at each menstrual period. While the cervix is developed by the accumulation of fluid, its orifice becomes nearly obliterated, and a sense of fluctuation is easily perceived by the finger. The introduction of a catheter is followed by a gush of adhesive yellowish-white transparent fluid, when the distended cervix collapses, and the patient experiences great relief. It is a condition that occurs only in women of a lymphatic temperament, and who have never had any children. Its treatment consists in incising the cervix at the commissures of the os, by which the fluid is evacuated, and prevented from collecting again.

*Inflammation of the uterus.* An interesting case of inflammation of the uterus, terminating in abscess which communicated with the rectum, is related by Dr. F. Bird.§ The patient was 37 years old; her health had been good until she married at the age of 34. Soon afterwards she began to experience pain in the pelvis, increased by micturition and defecation. A sudden discharge of pus from the rectum was followed by considerable relief to the pain, but during the succeeding two years she suffered from purulent diarrhea, though on the whole her general health improved during this period. At the end of this time, however, all her previous symptoms returned with greatly increased severity; menorrhagia, too, came on, and the patient sank. The uterus was firmly attached by its upper and posterior part to the rectum, and the fundus uteri was thrice the natural size. This enlargement of the fundus was produced by an abscess in its parietes, containing an ounce of thick pus; and communicating with the rectum by a sinus, that was wide enough to admit a probe. No communication existed between the uterine cavity and the abscess. A case is likewise recorded by Dr. Lever,|| in which the walls of the uterus contained a deposit of purulent-looking fluid, about the size of a kidney-bean. [The only cases with which Dr. Bird appears to be acquainted, are those related by Boivin and Dugés. Mention of others will be found in Busch, Geschlechtsleben des Weibes, Bd. iii, S. 701; Meissner's Forschungen, Bd. v, S. 167; and Meissner's Frauenzimmerkrankheiten, Bd. i, S. 928.]

Dr. Lever¶ notices that after the cure of *inflammation of the os and cervix uteri*, an intolerable pruritus sometimes continues, which may give rise to a state bordering on nymphomania. This is usually attended with slight leuchorrheal discharge, and

* Lond. and Edinb. Monthly Journal, July 1843, p. 660.

‡ Gaz. des Hôpitaux, Jan. 10, 1843.

§ Lancet, Jan. 28, 1843.

|| Op. cit., p. 27.

¶ Ib., p. 77.

sometimes considerable redness of the vagina is induced by the patient's rubbing herself to relieve the itching. On an examination, the finger is often stained with blood; and on introducing the speculum the os uteri is seen to be beset with numerous small granules, not unlike millet-seeds, white, soft, seemingly vesicular, generally in great numbers, and consisting of the enlarged glands of the os and cervix uteri. If there be much local tenderness, cupping or leeching may be necessary; if not, it will be sufficient to attend to the general health, and to employ injections of a drachm of sulphate of iron to a pint of water; or of five grains of nitrate of silver to an ounce of water.

*Ulceration of the cervix uteri.* M. Gosselin* denies that any peculiar symptomatic value is to be attached in the great majority of cases to ulcers of the cervix uteri. He regards them as forming only a very accessory part of the disease, which really consists in inflammation of the uterine tissue, especially of its lining membrane, against which, and not against the mere ulceration, remedial efforts should be directed. He details several observations, the purport of which is to prove that ulcerations of the cervix uteri give rise to no special symptoms, that they merely indicate the existence of a fully developed chronic metritis; and, except in syphilitic cases, need no peculiar attention. His experience also leads him to the important practical conclusion that local caustics have been far too extensively employed, that treatment should be directed to the uterine engorgement and consequent uterine catarrh, not merely to the cure of the ulcerations. It is evident that the ulcers may be cured by caustic, and yet the uterine catarrh and the general symptoms continue unchanged; since the application of caustics to the cervix uteri can have no influence on the diseased lining membrane of the organ. In the treatment of ulcers of the cervix uteri, M. Jobert† prefers the actual cautery to any forms of caustic. Having introduced an ivory speculum into the vagina, he employs cauterizing irons with a small button at the top. Their application does not excite the slightest pain, nor is it ever followed by any serious symptom, while it often gives most speedy relief. It has the advantage of not being followed by severe pain, such as succeeds the use of the nitrate of silver, while its action never extends beyond the spot to which it is applied; and in this respect it is preferable to the caustic potash. M. Jobert uses it especially in those large fungoid ulcerations, attended with frequent hemorrhage and neuralgic pains, which generally coincide with hypertrophy and ramollissement of the cervix.

*Polypus uteri.* M. Marchal de Calvi has published a very elaborate paper‡ on the spontaneous cure of this affection, founded on twenty-four observations, of which only one came under his own notice. It appears that this is effected in different ways. Sometimes the pedicle of the polypus is strangulated by the os uteri, and gangrene taking place it is thus detached; or the action of the uterus separates it during some of the violent attacks of hemorrhage to which patients suffering from this affection are liable; or the pedicle becomes gradually elongated, and is at last broken by the weight of the tumour; or it becomes detached during labour either by the head of the child pressing on it, or by the action of the uterine contractions.

M. Lisfranc§ advocates the excision of polypi in almost all cases, as preferable to the employment of the ligature; and states that he has seen serious hemorrhage succeed its performance only twice in one hundred and sixty-five times. Still in any case where the patient is already extremely exhausted by frequently repeated hemorrhage, it may be the more prudent plan to employ the ligature. The wound which follows the excision is sometimes very indisposed to heal, and may, unless attended to, degenerate into a troublesome ulceration. It is not, however, at all serious in its character, but yields readily enough to cauterization.

*Extirpation of the uterus.* The son of Professor Langenbeck, of Göttingen, has published‖ an account of the post-mortem examination of a person, whose uterus had been extirpated by his father in 1813, and who survived the operation twenty-six years. Considerable prolapse of the uterus existed; a circumstance which facilitated the operation of enucleating the uterus without wounding the peritoneum. Doubt

* Archives Gén. de Médecine, Juin 1843. † Gaz. des Hôpitaux, Mars 15, 1843.

‡ Annales de la Chirurgie, Août 1843.

§ Clinique Chirurgicale de l'Hôpital de la Pitié, t. iii, p. 210, and Gaz. des Hôpitaux, Oct. 18, 1842.

‖ De totius uteri extirpatione, auctore M. Langenbeck; Gottingæ, 1842.

has been expressed by many, as to whether Langenbeck had completely extirpated the organ; but the description of the post-mortem examination, and the drawings by which the dissertation is illustrated, show that he had completely succeeded. The dissertation contains, also, historical details with reference to the operation, and displays much diligence and research. A fatal case of extirpation of the uterus is recorded by Mr. Heath, of Manchester;* in which the operation was commenced under the impression that the body to be removed was an ovarian tumour.

DISEASES OF THE UTERINE APPENDAGES.

*Diseases of the ovaria. Tumours and dropsy of the ovaria.* Dr. A. Kilgour† endeavours to point out the signs distinguishing solid tumours of the ovaries associated with effusion into the peritoneum, from ovarian dropsy. In the diagnosis between these two a point of considerable practical importance is involved, since while it is generally thought undesirable to tap an ovarian cyst, considerable benefit often results from evacuating the fluid from the peritoneum in cases of solid ovarian tumours. Dr. Kilgour details three cases fully, and gives a tabular view of twenty-five others, which illustrate his views. His remarks for the most part substantiate the opinions of Curveilhier and Rostan, without adding to them anything very novel. He notices especially the gravitation of ascitic fluid, owing to which the small intestines are generally found yielding a clear sound about the umbilical region. Solid tumours of the ovary usually have a remarkable mobility, and their different surfaces will often come under the hand of the examiner, while in dropsy of the ovaries the swelling is fixed and immoveable. In ovarian dropsy, too, the neck of the uterus is drawn upwards, sometimes to so great an extent as not to be within reach of the finger when introduced into the vagina.

Two cases of accidental rupture of an ovarian cyst are recorded by Dr. Schlesier,‡ and Mr. Domville.§ In the first case the patient survived, but as her health gradually improved, the fluid collected again, and the abdomen, which had collapsed on the receipt of the blow, eventually regained its former size. Mr. Domville's patient died in a few hours. The ovarian sac was found extensively connected by old adhesions with the viscera and the abdominal parietes, although no inflammatory symptoms had existed during life. Other cases are related illustrative of the difficulty of determining, beforehand, whether an ovarian sac is adherent to surrounding parts, and on this difficulty Mr. Domville founds objections to the operation of extirpation of the ovary.

Dr. Ollenroth has published an essay, entitled, 'Ovarian Dropsy Curable,'‖ in which he advocates an operative proceeding, of a rather novel character, but somewhat analogous to the method first suggested by Le Dran, and afterwards by A. G. Richter. It consists in puncturing the abdomen in the ordinary manner, but, instead of completely emptying the cyst, allowing of the escape of only a portion of its contents, as, for instance, a fourth, or a third. The canula is not, however, to be then withdrawn, but merely closed by a stopper adapted to it, and a bandage that will easily admit of being tightened, is to be placed round the abdomen. The stopper is to be removed, and some of the fluid to be permitted to escape once or twice a day; by which means, the cyst is gradually brought to contract, and its walls adhering to each other, the patient becomes perfectly cured. This plan has been practised with success by Dr. Ollenroth, in one instance. The patient was 49½ years old, had suffered from ovarian dropsy for eight years; had undergone paracentesis seven times; on each occasion at shorter intervals, and her general health was completely broken down, when this plan of gradually evacuating the cyst was resorted to. The operation was performed on May 12th; and on the four succeeding days the stopper was removed from the canula twice every day; from the 16th of May to the 6th of June it was removed once daily. The fluid, which at first was yellowish, tenacious, and such as is usually contained in ovarian cysts, became by degrees puriform, and horribly offensive; but the patient's health appears to have gone on improving. On the 6th

* Med. Gazette, Dec. 9, 1843.
† London and Edinburgh Monthly Journal, June 1843.
‡ Casper's Wochenschrift, Aug. 5, 1843.
§ Med. Gazette, Nov. 25, 1843.
‖ Die Heilbarkeit der Eierstocks-wassersucht; Berlin, 1843.

of June no fluid escaped from the canula, which was removed on the 8th; and on the 12th, the wound made by the puncture was perfectly healed. The abdomen was, at that time quite collapsed, nor has it subsequently enlarged, and after a lapse of more than two years the person continues in good health, and capable of very considerable bodily exertion, without any indication of her former disease having reappeared. Very interesting historical details, with reference to the various operations that have been attempted for the cure of ovarian dropsy, occupy a great part of the pamphlet, but no other instance in which Dr. Ollenroth has tried this method is recorded. He employed it, however, in a case of chronic ascites, for which the patient had been tapped twice; her health deteriorating rapidly after each puncture. This new mode of procedure gave her temporary relief; and her death, which took place 31 days afterwards, does not appear to have been accelerated by it.

Considerable interest has been excited by an attempt to revive the operation of gastronomy, for the removal of diseased and dropsical ovaria. This attempt originated with Dr. Clay,* of Manchester, four of whose patients survived the operation, two sank under it, in one of whom it was left unfinished. Mr. H. D. Walne† has performed it in five cases; thrice successfully; one patient died, and in one the operation was abandoned, on account of difficulties which presented themselves, after it had been commenced. Successful cases have occurred to Mr. Southam,‡ Dr. F. Bird,§ Mr. Lane,|| and Dr. J. Atlee,¶ who is said to have removed both ovaries. Fatal results followed the operations of Messrs. Key, Cooper, and Greenhow:** and Dr. Foltz, in his report of Dr. Atlee's case, mentions an instance, hitherto unknown, of the fatal issue of the operation, in the hands of Dr. M'Dowall. It appears, that having laid open the abdomen of a woman, with the intention of removing a diseased ovarium, he found that the supposed tumour was formed by a mass of intestines agglutinated together by adhesions. The wound was closed, but the patient died of the effects of the operation. Mr. Heath's fatal case, already alluded to, at page 547, is another instance of the difficulties in the way of forming a correct diagnosis. Mr. Walne was the first to suggest making a small exploratory incision, with the view of ascertaining the presence or absence of adhesions, before laying open the whole cavity of the abdomen. In other respects, however, his operation coincided, as did the others, with the exception of Dr. Bird's, with that practised by Mr. Lizars, and involves the laying open the whole, or nearly the whole, extent of the abdominal cavity. Dr. F. Bird, anxious to avoid what he conceives to be an unnecessary exposure of the peritoneal cavity, has performed what may be regarded as an intermediate operation, between the major, as performed by Mr. Lizars, and the minor, as practised by Mr. Jeaffreson. He makes an opening, from four to six inches long into the abdomen, then punctures the tumour, and having evacuated its fluid contents, ties and divides the pedicle. This practice, which is similar to that attempted in one instance by Dr. Dohlhoff, of Magdeburgh, permits the introduction of the hand into the abdomen, and, consequently, the division of adhesions, and the secure application of ligatures to the pedicle; points which give it, in the opinion of Dr. Bird, the superiority over Mr. Jeaffreson's operation.††

* In a pamphlet, reprinted from vol. vii of the Med. Times; also in the same journ. for Oct. 7, 1843.

† His successful cases are reprinted from the Med. Gazette; the particulars of his unsuccessful cases have just been published in the Medical Gazette for Feb. 23 and March 1, 1844.

‡ Med. Gazette, Nov. 17 and 24, 1843. § Ibid., August 18 and Dec. 29, 1843.

|| The particulars of Mr. Lane's case are not yet published; some interruption having occurred to the convalescence of the patient; on which account he has delayed its public announcement till he can speak with certainty of the result.

¶ Reported by Dr. Foltz, who, however, did not witness the operation, in New York Journal of Medicine, for Sept. 1843.

** Mr. Key's case is contained in Guy's Hospital Reports, Oct. 1848; the other two were read at the Medico-Chirurgical Society, and are reported in Medical Gazette, Jan. 19, 1844.

†† The arguments for and against the operation, and the results hitherto obtained by its performance, are discussed in the Br. and For. Med. Rev., Oct. 1843 and Jan. 1844. In his enumeration of cases in which the operation was not completed, the writer of that article has omitted to notice the two cases recorded by Dr. Gooch, in his Account of some of the most important Diseases of Women, pp. 222-23, in which, after the abdomen was laid open, it was discovered that no tumour existed. In both these cases, the patients survived.

### III.—ON THE PROGRESS OF KNOWLEDGE WITH REFERENCE TO THE DISEASES OF CHILDREN.

### 1. Diseases of the Fœtus.

Dr. Keiler,* and M. Souty,† each detail a case of singular *congenital disease of the skin*, somewhat resembling icthyosis. In Dr. Keiler's case, the mother had had three healthy children, and after suffering severe abdominal pain from the third month of her fourth pregnancy, was confined, in the seventh month, of a child, who cried feebly, and survived only twelve hours. The skin of its head, face, and whole body was hard, thickened, of a structure resembling cartilage, and rent by deep irregular fissures. Tubercular prominences, without apertures, existed in the situation of the nose and ears, red fleshy masses occupied the orbits, and the genitals were represented by a mere knob. The skin of the trunk and extremities was less affected than that of the head and face. The mother, who was perfectly free from any syphilitic taint, subsequently gave birth to a healthy child. In M. Souty's case, it is probable, as Dr. Simpson observes, in some remarks upon it, appended to Dr. Keiler's paper, that the disease was originally of the same character, but had partly undergone the reparative process. The body of the child was traversed by reddish bands, inclosing irregular patches of scaly skin, in which the epidermis was hard, thick, and rough. The surface of these bands, or cicatrices, was covered with a thin, smooth pellicle, beneath which was the true skin with its texture unaltered. The nose and ears were in a rudimentary state, being deformed by the thickened epidermis: the eyes were natural in structure, though concealed by the everted eyelids; the disease of the skin having arrested the growth of the tarsal cartilages. The extremities were well formed, though disfigured by collections of matter beneath the skin. This child lived fifty hours.

A case of *croup in the fœtus* is related by Dr. Hayn.‡ He found, on examining the body of a child that died half an hour after birth, all the bronchi, both small as well as large, lined with a membrane, which was tubular in the larger branches, but quite blocked up the smaller ramifications. It was of a yellowish-green colour, and when examined under the microscope exactly resembled the exudation of croup.

### 2. Diseases peculiar to earliest Infancy.

*Injuries to the head in labour.* M. Danyau§ relates four cases in which fracture of the cranial bones occurred in the course of natural labour. In three cases the pelvis was contracted, in the fourth its dimensions were natural; and in none of the cases were the forceps employed. M. Danyau is of opinion that in the fourth case, in which the fracture could not have been the result of direct pressure, the parietal bones must have been forced over each other, and having bent to their utmost, then given way.

*Cephalæmatoma.* Dr. Churchill || has published a valuable digest of what has been written on this subject, but does not contribute to it any new matter. Dr. Zöhrer¶ of the foundling hospital at Vienna, regards it as a graver affection than the researches of Naegele, Feist, &c. represent it as being. He is accustomed to employ cold applications, to give calomel internally if there be much cerebral congestion; and it appears to be his invariable practice to open the swelling. It is evident from the directions he gives for the management of suppurating cephalæmatoma that the affection, under this plan of treatment, often becomes very serious. Dr. Doepp,** of the foundling hospital at St. Petersburgh, has met with 262 cases in 50,000 children: or 1 in 190. Of these, 64 per cent. were on the right, 31 per cent. on the left side of the head; and 5 per cent. on both sides. All were seated on the parietal bone except one, which occupied the squamous portion of the temporal, and two on the occipital bone. In his description of the disease he agrees with Naegele, Feist, and most recent observers, except in the opinion he expresses that the hard ring round the tumour

* London and Edinb. Monthly Journal, Aug. 1843.

† Bull. de l'Acad. de Méd., Oct. 1842.

‡ Medicinische Zeitung, Feb. 8, 1843.

§ Journal de Chirurgie, Jan. 1843.

|| Dublin Medical Journal, Nov. 1843.

¶ Med. Jahrb. des k.k. Oester. Staates, Dec. 1842.

** Oest. med. Wochenschr., Jan. 1, 1843.

usually disappears in four or five days. [The results of the writer's own observation too lead him to believe that Doepp is in this instance mistaken.] He confirms the opinion that the hard ring is formed by the edge of the coagulated blood effused beneath the pericranium; and states that it is only in rare cases, where the disease has been neglected, and the outer table of the skull has in consequence become absorbed, that the round edge of the inner table forms this ring. Three children died of the disease, two of whom were worn out by long-continued suppuration; one died of inflammation of the membranes of the brain, induced by cauterizing the tumour with lunar caustic. If absorption of the blood do not seem to be commencing after fomentations have been employed for a fortnight, M. Doepp is accustomed to make an incision, and to treat the wound with water-dressing. [In this interference he is probably premature; absorption does not always become perceptible within the first fortnight, and Feist's* experience makes it manifest that the best treatment of cephalæmatoma is to let it alone.]

*Infantile erysipelas.* Dr. Martin, of Jena†, endeavours to prove that this disease does not differ in any important respect from erysipelas in the adult, though it presents some peculiarities in its phenomena, course, and results. The skin in the infant has more of a velvety appearance, and is not so shining as in the adult; vesication seldom takes place, but small collections of pus in the subcutaneous cellular tissue are very frequent, and occur simultaneously in different parts; by no means confining themselves to those where the erysipelas originally commenced. Its wandering character is another distinguishing feature of the disease in the child, as also is its tendency to gangrene. It prevails sometimes epidemically; and did so at Jena at a season when erysipelas was frequently met with in the adult, and when phlebitis often followed venesection, an accident which occurred at that period in almost all the cases of puerperal fever in the lying-in hospital, in which blood was abstracted from the arm. Dr. Friebe‡ describes a peculiar form of erysipelatous inflammation of the neighbourhood of the navel which occurs in early infancy, and for which he proposes the name of *omphalitis exsudativa.* He has seen it thrice, coming on three weeks after birth, commencing about the umbilicus, and attended with infiltration of lymph or pus into the subcutaneous cellular tissue, and deposits of a similar matter in the partially obliterated umbilical vessels. It proves fatal in the course of forty-eight hours, without having extended further than three fingers' breadth around the navel; rapidly increasing exhaustion and occasional convulsive symptoms being the chief constitutional phenomena to which it gives rise. He inclines to regard it as a variety of partial *induratio telæ cellulosæ*, on account of the cachectic character of the children in whom it occurred, as well as the erysipelatous nature of the inflammation; while the fact of the umbilical vessels being in part converted into fibrous cords before the commencement of the disease induces him to think that it does not arise from phlebitis.

*Aphthæ and muguet.* Dr. Oesterlein§ has investigated the nature of the false membranes formed in cases of aphthæ and muguet, and has submitted them to microscopic examination. Under a moderate power they appear as an agglomeration of roundish corpuscules, intermixed with cylindrical fibres. Under a high power these fibres appear jointed and hollow; a transverse partition dividing the canal at each joint. Many of these fibrillæ contain sporules, which are round granules 0·0008 to 0·0012 of a Paris inch in diameter: many too beset the exterior of the fibrils, or float free around. It appears, however, that these structures are seen only at the period of the fullest development of the aphthæ, and Dr. Oesterlein's opinion is that the essence of the disease consists in an inflammatory condition of the mucous membrane of the mouth, which tends very rapidly to exudation, but that the formation of confervæ is

* Ueber die Kopfblutgeschwulst der Neugebornen; Mainz, 1839, 4to.

† Neue Zeitschrift für Geburtskunde, xiii Band, S. 402.

‡ Journal für Kinderkrankheiten, Oct. 1843.

§ Allg. Med. central Zeitung, 30 Nov. 1842. Dr. Gruby, whose researches were carried on at a period anterior to that included in this Report, holds the opposite opinion. See Clin. des Hôpitaux des Enfans, Sept. 1842. He regards the parasitic deposit or aphthophytes as the essential part of the disease: ulcerations, &c. as accidental complications. See also the observation of Dr. Berg of Stockholm, in the same journal for August, 1842.

accidental. In spite of many attempts to transplant the confervæ to other animal tissues or fluids he could never succeed in so doing. Dr. Effenberger* describes the muguet as he observed it during six months' residence in the foundling hospital at Vienna. It is there a very frequent and very fatal malady. The disease is probably in a great measure owing to the great crowding of the children; having been diminished since improved ventilation has been resorted to; but there does not seem to be any reason for regarding it as of a contagious nature. Its appearance was often announced by the occurrence of an erythematous eruption on the buttocks, sometimes of a papular, at other times of a vesicular character; and it was attended with diarrhea that frequently became very obstinate. The papillæ of the tongue became enlarged and elevated, and then covered with a white deposit which extended by degrees over the whole interior of the mouth. In bad cases deep ulcers appeared on the arch of the palate, which impaired the voice and rendered deglutition very difficult. The more dangerous cases too were marked by the increasing severity of the diarrhea, which exhausted the patient and occasioned death. After death the gums were found ulcerated; and the mucous membrane of the stomach red, soft, thickened and excoriated. The follicles of the small intestines were tumefied and sometimes ulcerated, and those of the large intestines, as well as the mesenteric glands, were swollen. The blood was fluid and the lungs were anæmic, except in those cases in which pneumonia had existed, when they were more or less extensively hepatized. M. Trousseau† remarks on the difference between idiopathic and symptomatic muguet. The latter is an accidental complication of various grave maladies, such as occur in foundling hospitals. The idiopathic form comes on without any more serious symptom than some heat of skin and fretfulness for a few hours before its eruption; and afterwards there is but slight derangement of the health, and perhaps a little diarrhea. In either form the same local applications are proper, such as one part of hydrochloric acid to ten of water, or one of nitrate of silver to ten of water, or one of alum to three of honey.

### 3. Diseases of subsequent Childhood.

#### DISEASES OF THE BRAIN, NERVOUS SYSTEM, ETC.

*Acute hydrocephalus.* Very interesting statistical details with reference to the frequency of this disease, and the various circumstances which influence its occurrence, are contained in Dr. Bennett's essay on hydrocephalus.‡ He follows the data furnished by the tables of the registrar-general, from which he concludes that five per cent. of all deaths under fifteen years of age arise from this disease, and the Berlin bills of mortality yield a similar result. It further appears that residence in cities greatly increases the tendency to hydrocephalus, and this tendency increases in almost direct proportion to the degree of crowding of the population. The statement quoted from American writers that during the last thirty years hydrocephalus has increased twelvefold in New York, while the population has only quadrupled, is, however, probably exaggerated; for a disease may often appear to be more frequent simply owing to its becoming better known. Dr. Bennett shows that hydrocephalus is most frequent from two to seven years of age; that it is much more frequent and twenty per cent. more fatal in males than in females. MM. Rilliet and Barthez treat of the disease§ under the name of *tubercular meningitis*, reserving the term hydrocephalus acutus|| for those cases in which an accumulation of serum takes place rapidly but not as the result of inflammation in the cavities of the cranium or in the cerebral substance, [an attempt at strictly scientific nomenclature scarcely warranted by the present state of our knowledge.] They have not met with this effusion of serum as an idiopathic affection, but only as a secondary occurrence, and usually supervening in the course of the eruptive fevers.

Dr. Christie of Keith, and Dr. Wöniger of Hamburgh,¶ have published cases of the successful employment of iodine in the treatment of acute hydrocephalus. In both

* Oest. med. Wochenschr., March 4-11, 1843. † Gazette des Hôpitaux, May 18, 1843.
‡ The Causes, Nature, etc. of Acute Hydrocephalus. 8vo. London, 1843. Chap ii.
§ Traité des Maladies des Enfans, tom. iii, p. 492. || Ibid., t. i., p. 781.
¶ London and Edinburgh Monthly Journal, March 1843; Oppenheim's Zeitschrift für die gesammte Medicin, April 1843.

instances the disease had reached the paralytic stage, and all other means had been tried in vain. Dr. Christie gave a teaspoonful of a solution of pot. iodid., gr. xvj, iodine gr. iv, in water ℥j, every 4 hours, and rubbed the scalp with a weak ointment of the biniodide of mercury; and Dr. Wöniger dissolved ʒj of iodide of potassium in ℥ss of water, and gave at first 40, afterwards 50 drops every two hours. In Dr. Christie's case, the first indication of improvement appeared in 36 hours, in the other instance, not till after the lapse of three days, but in both, recovery was complete.

Under the name of *meningitis mesencephalica*, Dr Brockmann,* describes a peculiar form of cerebral affection, incidental to childhood. He wishes by the name that he applies to it, to express the fact that the membranes of the medulla oblongata and pons, are the chief seat of the disease. He first observed it as a sequela of scarlatina, but has since seen it occur as an idiopathic affection, and altogether has met with it in fourteen cases. It is sometimes associated with general affection of the brain; at other times it is uncomplicated, but though its earlier stages are not marked by any serious symptoms, it is a disease quite as dangerous as cerebral meningitis. In its uncomplicated form, the cerebrum generally is extremely pallid and bloodless, showing a striking contrast to the cerebellum, the veins of which are turgid with blood, while its substance also is often highly congested. This congestion increases in intensity about the central parts of the encephalon, and the membranes covering the pons and medulla oblongata, are found in a most marked state of inflammation, though no such condition is seen in the membranes of the cerebellum, but the inflamed membrane is perfectly isolated, and usually not more than a square inch in extent The inflammatory action produces effusion of fluid, sometimes to the extent of several ounces into the subarachnoid tissue: or sometimes a gelatinous matter is effused, or the exudation is of a purulent nature.

It is most frequent in previously healthy children, from three to ten years of age; and no marked symptom betrays the first period, or that of simple turgescence, which generally lasts one or two days. The child is dull and heavy, and the occiput is often hot, but the bowels are regular, there is no vomiting, intolerance of light, or disturbed sleep. The general dulness and vague complaints about the head increase in the inflammatory stage, besides which there are notable heat of the back of the head, retraction of the occiput such as comes on in the advanced stages of ordinary hydrocephalus, and convulsive twitchings of the limbs similar to the effect of slight electric shocks, recurring every few minutes while the patient is awake, but ceasing during sleep. The general febrile symptoms continue during the third stage, the pulse loses its frequency and fulness, but does not become either irregular or intermittent. The state of general disquietude subsides by degrees into a comatose condition, in which the occiput is more retracted than before, but in which there is no strabismus nor affection of the pupil; and the peculiar air of stupidity that characterizes hydrocephalic patients is wanting. Two pathognomonic symptoms, however, announce the occurrence of this, the stage of effusion. One of these is deafness; the other is difficult articulation, and difficulty in moving the tongue; both of which occur at the same time, probably from paralysis of the motor nerves of the tongue. Usually the deafness and the affection of the tongue come on suddenly; sometimes the child awakes from quiet sleep with them. They are the earliest and surest signs of effusion. This stage ends sometimes in three days, sometimes not till after a fortnight in fatal paralysis; which is ushered in by various singular nervous phenomena; as sudden pauses in the respiration, or equally sudden syncope. Sometimes, however, these anomalous symptoms subside, and the patients gradually recover; indeed until the paralytic stage is fully established, recovery may be considered possible.

The disease is to be treated by depletion, which in most cases should be local, by cold to the head, and the free administration of calomel. Calomel may still be employed in the stage of effusion; but ammonia should also be used; the strength must be supported, and wine may be required. The most powerful counter-irritants, as large blisters, or the actual cautery, are also sometimes useful.

*Tubercle of the brain.* Dr. Hennis Green† has published some observations on this subject accompanied with a tabular view of thirty cases. His observations were

* Holscher's Annalen, Bd. ii, S. 678, and Bd. iii, S. 3. † Med.-Chir. Trans., vol. xxv, p. 192.

made at the Hôpital des Enfans Malades at Paris; and the large number of scrofulous cases in that institution will probably account for his estimate of the frequency of tubercular deposits in the brain being as high as one in fifty. The brief remarks which he makes on the morbid anatomy of the affection do not add anything to our previous knowledge, but the fact that tubercle was in no instance confined to the brain is interesting. In four of the cases no symptoms of cerebral tubercle existed during life, in four others the indications of its existence were very slight. In the remaining cases a chronic stage existed, in which the disease was betokened either by,—

1st. Headach, followed by various lesions of sensibility, or of muscular power; or,

2d. By convulsions, or epilepsy, gradually terminating in paralysis; or,

3d. By paralysis of one of the limbs.

He agrees with Bredow,* who has given the history of five cases of tubercle of the brain in regarding headach as the most important symptom, it having existed in seventeen cases. Next in frequency are partial or general convulsions, epilepsy, paralysis; or contraction of certain muscles or limbs, remarkable change of temper, and amaurosis.

In seventeen of the thirty cases, death took place from the supervention of some acute disease, as measles, variola or pneumonia, or the patients died in the course of phthisis. In thirteen cases, the chronic stage was succeeded by acute symptoms, which resembled more or less those of acute hydrocephalus. Dr. Green alludes to the anomalous character of the symptoms in some of these cases, and suggests that in those cases where hydrocephalus has appeared to run an anomalous course, the real cause of these peculiarities may have been the presence of tubercle in the brain. The observations of MM. Rilliet and Barthez,† agree for the most part with those of Dr. Green, though they appear to attach greater importance to the occurrence of convulsions than to the existence of headach, as a symptom of cerebral tubercle, and regard it as a more frequent symptom. In those cases where tubercular deposit in the substance of the brain or its membranes is unattended with symptoms betokening its existence, it will be found usually to coincide with far advanced tubercular disease in other parts, while the reverse is usually the case in those instances where marked cerebral disorder has existed. The various indications too of that obscure form of tubercular meningitis which often occurs towards the close of life in phthisical children are carefully detailed. An interesting case of tubercle of the brain in a boy aged two years, is recorded by Mr. Dunn.‡

*Hemorrhage into the brain, or its membranes.* Since the appearance of M. Becquerel's paper on this subject,§ it has been carefully investigated by M. Legendre‖ and MM. Rilliet and Barthez,¶ who have arrived at very similar results. Effusion of blood into the cavity of the arachnoid, appears to be the most common form of cerebral hemorrhage. Pure, unchanged blood, however, is but seldom found there; for it undergoes alteration very rapidly; the crassamentum separating from the serum and assuming in the course of time a membranous appearance. This membranous production forms first on the parietal layer of the arachnoid, afterwards on its visceral layer, thus forming a kind of cyst which is rarely empty, but generally contains a serous fluid in much larger quantity than that of the blood originally effused, and occasionally amounting to one or two pints. This accumulation of serum distends the yielding cranial walls of the child, and gives rise to a peculiar form of chronic hydrocephalus, which it is not always easy to distinguish from the same affection arising from other causes. Usually, however, the enlargement of the head takes place more rapidly than in ordinary chronic hydrocephalus, and the patients in which meningeal apoplexy occurs are always under two years of age, while chronic hydrocephalus from other causes often comes on at a later period of life. These cysts do not continue unaltered; but if the patient should survive, their contents become

* Med. Zeitung, Dec. 8, 1842.
† Lib. cit., tome iii, p. 552.
‡ Med. Chir. Trans., vol. xxv, p. 209.
§ Clin. des Hôpitaux des Enfans, 15 Avril, 1842.
‖ Revue Médicale, Dec. 1842, and Feb. and March, 1843.
¶ Lib. cit., tome ii, p. 29.

gradually absorbed, and the two layers being thus brought into contact adhere at different points, and form a multilocular cyst, or several small cysts. Even this state does not continue; but the walls of the cyst, which sometimes have a stratified arrangement, indicating that several successive effusions of blood have taken place, become gradually converted into an opaque firm membrane, having a pearly lustre, and closely resembling the dura mater in appearance. The symptoms of meningeal apoplexy, or of that much rarer form of cerebral hemorrhage, in which the blood is effused into the substance of the brain, are extremely obscure, so much so, indeed, as to render their diagnosis from other cerebral affections extremely difficult; and they are hardly ever attended by those apoplectic symptoms which denote such occurrences in the adult. A case, indeed, is related by Dr. Allier,* in which a child aged two years, after being exposed to the sun, was seized with violent convulsions, affecting chiefly the right side, and followed by coma, and paralysis of the right side. The coma subsided within a fortnight; the paralysis abated by degrees, and finally disappeared at the end of three months. This case is regarded by Dr. Allier as having been one of cerebral hemorrhage; [it must, however, be borne in mind, that while symptoms of just this kind have been observed, where no trace of hemorrhage was found after death, no case in which a post-mortem examination revealed the existence of cerebral hemorrhage has been attended in the acute state by paralytic symptoms.]

*Chronic hydrocephalus.* Dr. Whitney,† having had his attention called to the subject of cerebral auscultation by the researches of Dr. Fisher, in 1838, has since frequently employed it, and believes that a recourse to it will aid in the diagnosis of various diseases of the brain. In chronic hydrocephalus he has found that a cephalic bellows-sound constitutes one very prominent symptom; the existence of which he has substantiated in five cases. The sound is heard most distinctly in those situations where ossification of the skull is incomplete. It is a coarse, rough, and rasping sound, synchronous with the pulsations of the brain, and movements of the circulatory apparatus. In one instance, in which the brain was punctured, the sound was modified by the evacuation of the fluid, and became a low and indistinct murmur, but regained much of its former character as the fluid reaccumulated. Dr. J. R. Smyth‡ likewise notices the existence of a cerebral murmur as symptomatic of the first stage of chronic hydrocephalus, and states that his discovery of the phenomenon was made without any knowledge of its having been previously observed by Dr. Fisher.

Dr. Hannay§ relates a case of chronic hydrocephalus, in which he believes that recovery was in a great measure due to the employment of an ipecacuanha liniment to the scalp. The formula he adopts is the following:

℞ Ipecac. pulv. ʒij.

Olei oliv., ʒij.

Adipis, ℥ss. M.

The employment of this liniment three or four times daily is followed in about thirty-six hours by a papular and vesicular eruption. This form of counter-irritation was extremely useful in the case alluded to, and Dr. Hannay is of opinion that since chronic hydrocephalus often succeeds to the suppression of eruptions on the scalp, the use of this counter-irritant, the effects of which are much more manageable than those of tartar-emetic ointment, will in such cases prove very beneficial.

M. Trousseau‖ insists on the importance of watching the effects of compression of the head, and relates a case in which the neglect of this precaution was followed by laceration of the brain, the escape of the fluid by the nares, and the patient's death. It was found that the resistance of the bandage had caused the fluid to act exclusively on the base of the brain, and that the ethmoid bone had thus been dissevered from its connexions.

In order to ensure a firm and equal pressure on the head, M. Trousseau is accustomed to employ the following method. The hair being clipped as short as possible,

* La Clin. des Hôp. des Enfans, Oct. 15, 1842.
† Amer. Journ. of Med. Sciences, Oct. 1843.
‡ Medical Gazette, May 19, 1843.
§ Edinb. Med. and Surg. Journal, Oct. 1843.
‖ Journal de Médecine, Avril 1843.

he applies strips of diachylon plaster four lines broad. 1st. From each mastoid process to the outer part of the orbit of the opposite side. 2d. From the hair at the back of the neck, along the longitudinal suture to the root of the nose. 3d. Across the whole head, in such a manner that the different strips shall cross each other at the vertex. 4th. A strip is cut, long enough to go thrice round the head. Its first turn passes above the eyebrows, above the ears, and a little below the occipital protuberances, so that the ends of all the other strips shall project about three lines below the circular strip. These ends are next to be doubled up on the circular strip, and its remaining two turns are then to be passed over them just in the same direction as the first turn.

In a case of chronic hydrocephalus, in a child aged 3 years and 4 months, Dr. Whitney* punctured the brain with success. Nine ounces of fluid were withdrawn by the first operation, and five ounces more on its repetition three weeks afterwards. No untoward symptom followed either operation, and the child's recovery appears to have been complete.

Two cases of the unsuccessful employment of puncture of the brain are recorded by Professor Wutzer and Mr. Butcher.† In the first case it had been supposed that the fluid was exterior to the brain; but no fluid following a slight puncture, it was not thought warrantable to plunge the trocar deeper. Six days afterwards the child, aged 7 months, died in convulsions; and 22 oz. of fluid were found in the ventricles. The subject of the second case was 16 months old; the previous symptoms had been severe. Tapping was twice performed; the second time four weeks after the first. The child appeared benefited by the operations, but died in convulsions eleven weeks after the first puncture, owing in great measure, in Mr. Butcher's opinion, to the mother having given it wine.

*Paralysis.* The writer of this Report has published a paper on the subject of paralysis in infancy and childhood.‡ He attempts a division of it into three forms, according as the affection is congenital; or succeeds to symptoms of cerebral disturbance; or comes on without any previous indication of disorder of the brain. It usually presents the form of hemiplegia, and the leg is affected by it oftener than the arm. Sensation is not impaired; occasionally, indeed, it seems to be morbidly increased. The first variety is usually associated with imperfect nutrition of the affected limbs, and, as might be expected, is incurable. Cases of the second class, for the most part, do well eventually; they are often associated with constitutional disturbance dependent on the process of dentition, and require an alterative plan of treatment, and close attention to the state of the bowels. Cases of the third class occur in debilitated children; and sometimes succeed the eruptive fevers. They often run an extremely chronic course, and the patient's recovery is in many cases only partial; or, although his general health may become robust, the limb may continue powerless; in which cases it wastes, and becomes much smaller.

### DISEASES OF THE ORGANS OF RESPIRATION AND CIRCULATION; AND OF THEIR APPENDAGES.

*Stomatitis.* MM. Rilliet and Barthez§ notice the points of difference which distinguish between this disease and true cancrum oris. They remark upon its frequent occurrence, its tendency to prevail epidemically, its contagious nature, chronic course, and the absence of any disposition to become gangrenous; in all which respects it differs from cancrum oris. They mention, too, as characteristic of the disease, its tendency to the formation of false membrane. Dr. Hunt‖ applies the term cancrum oris to this affection; believing it to be only a milder form of gangrene of the cheek; [but the reasons for a contrary opinion, as stated by MM. Rilliet and Barthez, appear to be quite conclusive.] His chief object, however, is to recommend the internal administration of the chlorate of potash, which he believes to possess specific powers in controlling this affection. He is accustomed, after the use of a purgative if necessary, to give from 20 to 60 grains of the chlorate of potash, accord-

* Loc. cit. † Oesterr. Med. Wochenschr., Nov. 12, 1842; Dub. Journ. of Med. Science, March 1843.
‡ Medical Gazette, Sept. 8, 1843. § Lib. cit., tome i, p. 260, and tome ii, p. 149.
‖ Med.-Chir. Trans., vol. xxvi, p. 142.

ing to the age of the child, every twenty-four hours, dissolved in water. He states that its beneficial effect is often observed on the following day, almost always on the second; the disagreeable fetor soon lessens, the sores put on a healthy reparative action, the dribbling of saliva diminishes, and if there be mere ulceration it very speedily heals, if there be an eschar it soon separates, and the sore granulates kindly. MM. Rilliet and Barthez recommend the use of the chloride of lime in substance, as a local application to the foul ulcers of stomatitis, and advise that its employment should be continued for some time after a cure has apparently taken place.

*Diphtheritis.* M. Trousseau* remarks on the distinction between diphtheritis and true croup. He regards the former disease as being stomatitis in an aggravated and unusually severe form. Stomatitis in general is confined to the gums, which may continue affected by it for weeks or months; but it may also extend to the inside of the cheeks, which then become the seat of a pultaceous or membranous exudation. Sometimes, however, it is not limited to these situations; but false membranes appear on the uvula and tonsils, and give rise to the symptoms of angina maligna, when if prompt relief be not afforded, the disease involves the air-passages, and proves fatal. This occurrence is rare in the adult, but less uncommon in the child; and M. Trousseau speaks of a woman with an infant at her breast recently received under his care, the former of whom was suffering from the disease, limited to her gums; the infant caught it, the false membranes extended to its pharynx, and proved fatal. He alludes to its contagious character, and mentions some of the evidences of it adduced by M. Bretonneau. He states, also, that during an epidemic of diphtheritis, which came under his observation in the year 1828, the disease was confined to the gums in most adults, but involved the pharynx and larynx, and thus proved fatal to many children.

*Croup.* Dr. Ware† has written an elaborate paper on croup, which is valuable, as embodying the results of twenty-five years of observation. He is of opinion that two distinct diseases are generally included under the term croup, which, however, present considerable similarity to each other in their early symptoms. The more frequent of these affections is very mild in character, yields readily to remedial agents, and would probably subside, even though no treatment were adopted, while no neglect of curative measures would cause it to assume the grave characters of the second form. This second form, though much less frequent, is very violent; it is scarcely affected by any treatment, and almost invariably terminates fatally. In the course of twenty-five years, Dr. Ware has met with 131 cases of croup; the term croup being applied by him to every case in which there existed any embarrassment of the respiration, attended with affection of the voice, and a cough of a shrill, harsh, and ringing character. He subdivides these cases into the four classes of

| | Cases | Deaths |
|---|---|---|
| Membranous croup | 22 | 19 |
| Inflammatory | 18 | - |
| Spasmodic | 35 | - |
| Catarrhal | 56 | - |
| | 131 | 19 |

He distinguishes the first and second classes by referring to the first all cases in which there was reason to suppose that a false membrane had been formed, while the second differs from it only in the supposed absence of a false membrane. He relies for distinguishing the membranous form of croup less on the cough or voice than on the respiration. It is not loud, harsh, suffocative breathing, attended with great efforts and much loud coughing, creating great alarm, and calling at once for relief, but it is comparatively quiet and unobtrusive at the commencement of the disease, and unattended with apparent distress. There is only a little more effort than natural in inhaling and expiring the air, attended with slight dilatation of the nostrils, and a little whiz accompanying the passage of the air through the rima glottidis. Dr. Ware allows, however, that the characters of this form are but seldom met with so unmixed as in the above description; for paroxysms of violent spasmodic breathing occur,

* Gaz. des Hôpitaux, May 7, 1843. † New Eng. Quart. Journ. of Med. and Surgery, Oct. 184.

during which the respiration is accompanied with a loud râle, from the presence of mucus in the air passages. As the disease advances the muscular efforts at respiration become very strong, and the expiration is chiefly characterized by the amount of force used to expel the air.

Such are the symptoms enumerated by Dr. Ware as characteristic of the membranous form of croup. He acknowledges, however, that its symptoms are in a great measure common to the inflammatory form. His diagnosis between the two is founded on the presence of false membranes on the back of the fauces in membranous croup; an occurrence to which there are so few exceptions that of thirty-three cases of membranous croup, in which the fauces were examined, thirty-two presented false membranes on the tonsils, uvula, palate or pharynx. The diagnosis, however, was verified by a post-mortem examination only in fourteen cases, the others not having been examined after death; but in all of these fourteen, false membranes were found in the air-passages; and in one only they were present in the larynx, while no trace of them existed on the fauces.

In 45 cases of what he classes as the other forms of croup, three only presented a thin slight exudation on the tonsils; none a regular false membrane. Of these 45, 12 were of the second, 11 of the third, 22 of the fourth class.

He believes then that the membranous and inflammatory croups are different: 1st. Because the great preponderance of deaths over recoveries in the former, of recoveries over deaths in the latter; and the invariable presence of false membrane in the one, its invariable absence in the other, afford strong reasons for the supposition. 2d. Because, in his opinion, the effusion of false membrane requires an inflammatory action peculiar in kind rather than violent in degree. 3d. Because the phenomena which attend recovery from membranous croup are different in many respects from those which attend recovery from the simple inflammatory form.

Dr. Ware's third form of croup answers pretty nearly to laryngismus stridulus, [though he appears sometimes to have mixed up in his description some of the characteristics of true croup; thus falling into the error committed by Millar nearly a century ago.]

The fourth or catarrhal class includes cases which might more properly be called cases of threatening croup than referred to a separate form.

Mr. Copeman* recommends painting the exterior of the throat in cases of croup twice a day with a strong tincture of iodine. He conceives that it tends to prevent the formation of false membranes, and relates two cases in which its employment was apparently beneficial. M. Valleix† mentions in illustration of the good results produced by free vomiting in croup, that in 31 out of 53 cases antimony and ipecacuanha were employed in large doses as emetics, and 15 of the 31 recovered. In the remaining 22 their use was but sparingly resorted to, and only one recovered. It is probably as a powerful emetic that the sulphate of copper should be regarded; and Dr. Aberle‡ mentions the circumstances which should induce us to employ it in preference to the tartar emetic. These are a very depressed state of the system, in consequence of which the stomach has lost much of its natural irritability, and the occurrence of diarrhea as a consequence of the employment of antimony. Dr. Aberle speaks of having used it with success; and Dr. Schwabe§ talks of having given it in more than 50 cases. [Among these, however, were doubtless many in which none other symptoms existed than such as were premonitory of the disease, or as constitute the pseudo-croup of Guersent and other writers.]

The work of MM. Rilliet and Barthez contains an essay on tracheotomy, by M. Trousseau.‖ He attributes the fatal result which so often follows it to delay in its performance; and recommends that it should always be performed as soon as the presence of false membrane in the larynx has been ascertained. He prefers tracheotomy to laryngotomy; always introduces a canula into the trachea, but endeavours to dispense with its use as early as possible. He does not rely merely on the ope-

* Provincial Medical Journal, Aug. 12, 1843.

† Guide du Méd. practic., extracted in Bull. Gén. de Thér., Oct. 1843, p. 246.

‡ Oest. med. Jahrb., July and Sept. 1843.

§ Casper's Wochenschr., March 4, 1843.

‖ Lib. cit., tome i, p. 367.

ration, but sponges out the trachea with a sponge dipped in a solution of one part of nitrate of silver in five of water; and pours a few drops of a weaker solution into the air passages, a proceeding which he repeats several times during the first few days after the operation. Of 112 croup cases thus treated 27 recovered; or adding cases operated on by other surgeons we obtain a total of 150 cases and 39 recoveries, [a result probably not more favorable than is obtained by purely medical treatment.]

*Retro-pharyngeal abscess.* In a memoir on this affection M. Duparcque* mentions that 30 cases of it have been published since attention was first drawn to it by Dr. Abercrombie. Of these 30 cases 10 occurred in children from 11 months to $4\frac{1}{2}$ years of age. He relates the history of a delicate boy aged $4\frac{1}{2}$ years, who was attacked with febrile symptoms, attended with great cerebral disturbance, which was relieved by leeches. Difficult and painful deglutition, however, soon came on, accompanied with swelling of both sides of the neck, but especially obvious on the left side. These symptoms progressively increased, and became associated with extreme pain on pressure upon the larynx. At length there were signs of impending suffocation, with symptoms such as occur in an advanced stage of croup. On the eighth day after the commencement of the illness, during an attempt to examine the fauces, the patient made a sudden effort at vomiting, which was attended with the rejection of mucus, pus, and blood, and followed by great relief. The external swelling of the neck had disappeared, the child asked for food, and in a few days recovered. M. Duparcque regards this as having been a case of abscess behind the œsophagus, of which, and of retro-pharyngeal abscess, the symptoms are, 1. Swelling of the lateral parts of the neck. 2. Deglutition painful; at first difficult, afterwards impossible. 3. Laryngeal respiration, growing more and more difficult till it amounts to actual suffocation; which, 4, is rendered more imminent in certain postures. 5. Alteration of the voice. 6. Stiffness of the neck and immobility of the head.

The following symptoms are peculiar to abscess behind the œsophagus: 1. Severe pain produced even by moderate pressure on the larynx and upper part of the trachea. 2. The circumstance that such pressure produces entire suspension of respiration. 3. Displacement of the larynx forwards and to the right.

In order to be absolutely sure of the existence of this affection, the finger should be passed into the throat and quite behind the larynx, when the tumour of the œsophagus, if it exist, will at once be felt.

*Chronic enlargement of the tonsils.* M. Robert† treats of the results produced by this affection in childhood. The enlargement is never confined to one side, and occasionally it becomes very considerable; in which case it not merely affects the voice, as in the adult, but likewise, by its pressure on the Eustachian tubes, greatly impairs the sense of hearing. It further interferes with the passage of air by forcing up the velum, and hence children thus affected sleep with their mouth open. It also gives rise to a constant dry troublesome cough, imparts a nasal tone to the voice, and owing to air never passing through the nares, the nose does not become properly developed, but remains narrow, and the anterior part of the face thin. Its most serious result, however, is that lateral flattening of the chest, to which attention was first called by Dupuytren. M. Robert thinks that it is produced by the enlarged tonsils preventing the entrance of air, at each inspiration, in sufficient quantity to fill the vacuum in the chest, or to cause a pressure from within the lungs equivalent to the atmospheric pressure without. This deformity of the chest cannot exist without giving rise to dyspnea, palpitation, and the various results of interrupted respiration and circulation: hence children in whom it exists are usually pale, thin and weak.

The symptoms of enlargement of the tonsils usually make their appearance between the sixth month and the second year, and it is probable that the hypertrophy is due to the irritation of dentition. In further proof of this M. Robert mentions that he has seen evolution of the dens sapientiæ in the adult attended with inflammation and hypertrophy of the tonsils.

When once enlarged the hypertrophied tonsils never diminish in size; M. Robert therefore advises their excision. He suggests, moreover, various modes of diminishing the deformity of the chest, and describes different gymnastic exercises appropriate for this purpose.

* Annales d'Obstétrique, Dec. 1842.

† Bull. Gén. de Thérapeutique, May and July 1843.

*Pneumonia.* In their recent work* MM. Rilliet and Barthez have reproduced their former treatise on this subject, with such additions as more extended experience has enabled them to make. Their opinions, however, continue on all important points the same as they expressed in 1838. The writer of this report has made some observations on pneumonia in children.† The results of his post-mortem examinations confirm those of MM. Rilliet and Barthez, except in so far as lobar pneumonia appears to have come under his notice more frequently than it presented itself to those gentlemen. He remarks on the occurrence of idiopathic pneumonia as being more frequent than it would seem to be from the experience of French writers; and shows that it is not, as has been alleged, usually preceded by catarrhal symptoms, but comes on in the greater number of instances wholly independent of them. Its prevalence as well as its fatality are shown to be much greater during the first two years of life than at any subsequent period; while one attack of the disease renders the child prone to its recurrence. An attempt is made to describe its physical signs as well as its general symptoms: the former having hitherto been passed over with too little notice by English writers. The treatment recommended consists in depletion, which, however, is of less avail in the variety of pneumonia that is developed out of catarrh than in the purely idiopathic form. The indications for the employment of antimonial and mercurial preparations are pointed out; and the paper closes with remarks on the general management of the disease.

*Hooping-cough.* Dr. Aberle‡ has published a dissertation on hooping-cough, which contains, especially in the department of therapeutics, a most elaborate *résumé* of all that has been written on the subject. He embodies in it, likewise, the results of his father's experience during five successive epidemics at Salzburg: but does not throw any new light on the disease. He espouses the opinion that it depends on irritation and inflammation of the nervus vagus, but seems to have met with only one instance in which the appearances found after death betokened its existence. M. Schlesier§ notices the connexion between hooping-cough and measles, and details the particulars of an epidemic at Peitz, which illustrate the mutual relations of the two diseases. He employs his facts to support a theory, that, when the miasm of influenza is superadded to the contagion of measles, a *tertium quid*,—hooping-cough is the result. M. Trousseau|| notices the fact that an intercurrent febrile attack coming on in the course of hooping-cough always diminishes the disease, sometimes even effects its cure. He observes, too, that this modifying influence does not terminate on the patient's recovery from the fever, but that should the hooping-cough return, after its cessation, it will always be in a milder form. Both M. Jadelot,¶ and MM. Rilliet and Barthez** remark on the error of the opinion that hooping-cough tends to produce emphysema. They join in the observation that the single inspiration, being followed by a succession of rapid, forced expirations, would tend to diminish rather than increase emphysema, while its phenomena are the very opposite to those of suffocative bronchitis, in which the most laborious efforts are made to inspire a quantity of air sufficient for the preservation of life.

*Phthisis.* The treatise of MM. Rilliet and Barthez††, contains some very valuable observations on pulmonary and bronchial phthisis, but far too long to admit of a complete abstract being given in this Report. They notice that some of the physical signs, on which in the adult, we should lay considerable stress, as betokening the early stage of pulmonary phthisis, are of much less importance in the child: thus, for instance, harsh respiration and prolonged expiration are often met with in the infraclavicular region in children, wholly independent of any morbid state of the lungs. They likewise dwell upon the influence of tuberculated bronchial glands, in imparting an exaggerated character to all the physical signs of tubercle in the lungs. Many pages are devoted to the subject of bronchial phthisis, and their account of its symptoms is much more complete than any which has been published hitherto. No sign, perhaps, is of more importance, as indicating the existence of tubercle in the bronchial

* Lib. cit., tome i, p. 60.

† Report on the Pneumonia of Children, in British and Foreign Medical Review, April 1843.

‡ Tussis Convulsiva, etc.; Vindobonæ, 1843, 8vo.

§ Med. Zeitung, Oct. 4, 1843.

|| Gaz. Médicale, June 10, 1843.

¶ Gaz. des Hôpitaux, July 25, 1843.

** Lib. cit. tome ii, p. 217.

†† Ibid., tome iii, p. 164.

glands, than the variable character of the results of auscultation, while percussion invariably yields the same result. Sundry modifications of the voice, and a peculiar spasmodic cough, not unlike that of hooping-cough are likewise characteristic of bronchial phthisis.

*Diseases of the heart.* MM. Rilliet and Barthez* make some observations on endocarditis in childhood; but their remarks apply principally to chronic lesions of the valves. The writer of this Report has endeavoured to call attention† to the occurrence of endocarditis and pericarditis in childhood, as purely idiopathic affections coming on with comparatively trivial symptoms, but tending to produce permanent disease of the organ, and consequently, to lead to all those formidable and distressing symptoms that attend disease of the heart in the adult.

### DISEASES OF THE ABDOMINAL VISCERA.

*Inflammatory affections of the digestive canal.* MM. Rilliet and Barthez‡ have several long articles on this subject. They regard simple *gastritis* as being both unusual, and of minor importance in childhood. Their researches, too, with reference to *ramollissement* of the stomach have led them only to negative results, since they never observed it unattended with other diseases, nor did it in any case constitute the chief lesion. They found it oftenest in cases of meningitis, as also in other cerebral affections, in pneumonia complicated with head symptoms, in the eruptive fevers, and occasionally in inflammatory affections of the abdominal viscera.

They regard remittent fever, or typhoid fever as they term it, as being intimately connected with inflammation of the intestines. They conceive, however, that this inflammation partakes of a specific character, and differs in this respect from the simple enteritis of childhood. The ordinary form of enteritis in the child is that of erythematous inflammation; follicular enteritis is occasionally met with, and the appearances left by it in the intestines differ in no respect from those produced by typhoid fever. Simple follicular enteritis, however, is less serious, is unattended with inflammation of the mesenteric glands, while the symptoms attending it, and the circumstances under which it is developed, are altogether different from those which give rise to typhoid fever. Inflammation of the large intestines is much more frequent, and much more serious than that of the small intestines. [The statement of the writers, however, that in one out of every two post-mortem examinations that they made, traces were found of inflammation of the large intestines, must be received with due allowance for the scene of their observations having been the Hôpital des Enfans Malades.] Both the erythematous and the follicular form of inflammation of the large intestines are most severe about the termination of the colon, and are sometimes limited to that situation. The writers consider a classification of the symptoms, according to the part affected, to be almost impracticable, and therefore divide inflammation of the intestines into a simply acute, a typhoid, a dysenteric, and a chronic form; each of which may be primary or secondary. The greater number of their observations refer to the secondary forms; hence their detail of symptoms is of less value than their description of morbid appearances. Hence too, as the writers themselves remark, the disease has been more fatal under their hands than, probably, it might prove under other and more favorable circumstances.

*Tabes mesenterica.* The common error of regarding tabes mesenterica as a disease of frequent occurrence in childhood, is dwelt on by MM. Rilliet and Barthez.§ They show that various other diseases have been confounded with it, and that the large abdomen, natural to children, has been often regarded as an indication of mesenteric disease. They prove, by statistical data, that though slight tubercular deposit in the mesenteric glands is by no means unusual in tuberculous children, yet an extensive deposit is not met with more frequently than in one out of sixteen cases. Tabes mesenterica, too, appears to be very rare under three years of age, and to be most frequent between the ages of five and ten, beyond which period of life it occurs very seldom. Their remarks on the symptoms are valuable, but lead chiefly to a negative result, since they show that many signs, on which much reliance has been placed, are quite inconclusive, but do not furnish others more trustworthy in their room.

* Lib. cit., t. i, p. 217. † Med. Gazette, Aug. 18, 1843. ‡ Lib. cit., t. i, p. 452.
§ Lib. cit., tome iii, p. 406.

*Polypi of the rectum.* M. Bourgeois* and M. Gigon† notice the occurrence of polypi of the rectum as being more frequent in children than in adults. They do not resemble polypi in other parts, but are fleshy substances of a bright red colour, destitute of epidermis; their surface constantly oozing with blood, and attached to the intestine by a very delicate membranous pedicle, of a greyish colour, and about the diameter of a small quill. Their point of attachment varies from a few lines to a couple of inches within the rectum. If torn through they sometimes bleed; but this is not always the case. The occasional discharge of blood per anum is the symptom to which they most constantly give rise, and this hemorrhage is sometimes so considerable as to impair the patient's health. They do not by any means invariably protrude beyond the anus during the act of defecation. M. Bourgeois is of opinion, that in some cases nature effects their cure; the action of the sphincter detaching them, after which the pedicle shrivels. Their treatment, however, is very simple. M. Bourgeois is accustomed to twist them off from their pedicle with his fingers; and has seen no bad consequence result from it. M. Gigon, having seen troublesome hemorrhage from the pedicle, ties it, and then returns the polypus within the rectum, and in the course of two or three days it drops off.

*Nocturnal incontinence of urine.* Professor Romberg‡ proposes a new theory as to the cause of this troublesome infirmity. He denies that it arises from paralysis of the neck of the bladder; but attributes it rather to morbid sensibility of the orifice of the ureters. These spots are always much more sensitive than any other parts of the lining membrane of the bladder, and the irritation of the urine is felt still more keenly when this sensibility is morbidly increased. Reflex action of the musculus detrusor urinæ follows; especially in sleep, when the action of the will is suspended. He proposes, therefore, that children who suffer from this affection should be made to sleep on their belly, by which position the irritation of these two points by the urine is in a great measure avoided, while at the same time demulcents may be given to correct any irritant qualities of the urine.

FEVERS.

The treatise of Dr. Gregory§ on the eruptive fevers, as well as the work of MM. Rilliet and Barthez, contain many valuable observations on measles, scarlatina, and smallpox. The statistical details in Dr. Gregory's book are especially interesting, but it would not be possible to introduce numerical results into this Report.

*Measles.* Dr. Panck|| describes a mild, but extensive epidemic of measles, which broke out in May, 1842, in the Orphan House at Moscow. He relates several interesting facts, which show that while the establishment was outside the city, its inmates enjoyed a perfect immunity from epidemic diseases; but that since its removal to a locality in other respects more desirable, but situated within the city, the occurrence of epidemic diseases has been very frequent. The various exanthemata had prevailed extensively in the city during the previous winter; and in May the first case of measles appeared in the Orphan House. The epidemic ran its course in a month, during which time a hundred children, or one third of the inmates of the institution were attacked by it. Ninety-four of the children were pupils of the Orphan House, and only two of them died; a rate of mortality certainly very favorable. In many cases varicella or urticaria preceded the eruption of measles, while in other instances a kind of essera appeared on the hands and face for several hours, or even for some days before the measles showed themselves. When they appeared they were often attended with great drowsiness; but that symptom was not by any means unfavorable. The fever presented somewhat of a gastric type, and stomatitis was very frequent as an attendant on the disease, as well as one of its most frequent sequelæ. Diarrhœa occurred as a sequela in nearly half of the cases; and seemed to partake somewhat of a critical character, coming on in the stage of desquamation when the skin became dry, and the sweats, before very copious, altogether ceased. It was found to be the wisest plan not to attempt to check it; and, indeed, the disease in all its stages required hardly any active interference.

The author of this Report describes¶ an obscure and dangerous affection of the

* Bull. Gén. de Thérap., Oct. 1842, and Nov. 1843. † Annales d'Obstetrique, Avril 1843.
‡ Journal für Kinderkrankheiten, Oct. 1843. § Lectures on the Eruptive Fevers. 8vo, Lond. 1843.
|| Oppenheim's Zeitschrift, Oct. 1843. ¶ Medical Gazette, Aug. 25, 1843.

air-passages, which he had met with in some instances as a complication of measles, and which has not been often alluded to by former writers. It consisted in the supervention of stomatitis towards the decline of the eruption, associated with a tendency to the formation of false membranes, and the extension of the diphtheritic inflammation to the larynx. In some cases the croupy symptoms to which it gave rise were very obvious, while in other instances its existence was extremely obscure, and masked by the coexistence with it of inflammation of the lungs. It was usually betokened by great drowsiness, difficult deglutition, and alteration of the voice; which were more constant in their occurrence than croupy cough or stridulous breathing. The gums were generally spongy or ulcerated, the tongue was red and raw, and aphthous ulcers existed on its surface or on its edges; and the tonsils and soft palate were red, and coated more or less extensively with false membranes. The depressed state of the system contra-indicated the employment of depletion; and calomel and the other remedies of inflammatory croup were wholly inefficient. The writer expresses an opinion that the employment of local cauterization, as in cases of ordinary diphtheritis, would have been in several of the cases a more appropriate mode of treatment.

*Scarlatina.* Dr. Graves and Dr. Kennedy* furnish us with very interesting accounts of the scarlet fever recently epidemic in Dublin. The disease, which during the greater part of the present century had been very mild, assumed about the year 1831 a much more serious and fatal character, and continues still to retain it. Some of the cases were associated with severe head symptoms, and convulsions and coma came on at a very early period. In a second variety great irritability of the stomach and bowels existed from the very commencement, dependent, however, on cerebral irritation and congestion, not on any disease of the abdominal viscera. Cases of the third class went on favorably for eight or nine days, when the sore throat returned in an aggravated form, attended with great swelling of the parotid and submaxillary glands, and fever of a typhoid character, which terminated the patient's life. Dr. Kennedy's minute details respecting the affection of the throat are especially interesting. Dr. Watts† has detailed the particulars of a case in which croupy symptoms succeeded to an attack of scarlatina, owing to swelling and ulceration of the mucous membrane of the glottis. Tracheotomy was performed with complete success; but at the date of the report of the case, two months after the operation, respiration was not carried on through the larynx with sufficient freedom to warrant the removal of the canula, and the closure of the wound in the trachea. Dr. Bodenius‡ revives Peart's recommendation of the sesqui-carbonate of ammonia in the treatment of scarlet fever, and attributes to it almost the virtues of a specific. A similar recommendation is made by Dr. Rieken,§ though he forms a somewhat less exaggerated estimate of the virtues of the remedy. M. Godelle‖ advocates the employment of hydrochloric acid and of belladonna, as prophylactics against scarlatina. He attaches greater value to belladonna, but conceives that its preservative power exists only during the time that it is actually employed; and that it does not remove a person's susceptibility to the disease at subsequent times. The experiments of M. Stievenant¶ on its prophylactic virtues do not yield any positive results, but leave the question nearly as far from a solution as before; though, perhaps, they may be regarded as on the whole favorable to the virtues of the drug.

*Variola.* The anatomy of the smallpox pustule in the various stages of its progress has been studied with great care, and described with great minuteness, by MM. Rilliet and Barthez.** Both they and Dr. Stewardson, of Philadelphia,†† advocate the employment of plasters of mercurial ointment as a means of procuring abortion of the pustules, and consequently preventing pitting. Dr. Stewardson tried this plan extensively, in the Philadelphia hospital, and satisfied himself that it produced a decided effect if the plasters were applied before the third day of the eruption, and were allowed to remain undisturbed until the sixth. He doubts, however, whether

* Graves's Clinical Med., Lect. xxxiv; and Kennedy's Account of the Epidemic of Scarlatina, &c. A fuller account of their observations is contained in Br. & For. Med. Rev., Nos. XXXI & XXXIII.

† Med. Gaz., May 5, 1843.

‡ Ueber das kohlensäure Ammonium, etc.; Heidelberg, 1842.

§ Sur l'Emploi du Carbonate d'Ammoniaque, etc.; Bruxelles, 1843.

‖ Revue Médicale, Jan., Mars, Avril, 1843.

¶ Bull. de l'Acad. Royale de Méd., 15 Fev. 1843.

** Lib. cit., tome ii, p. 450.

†† American Journal of Medical Sciences, Jan. 1843.

the pitting will be always prevented by this measure. In two instances he applied plasters of other than mercurial ointment, but found that they produced no effect; he is of opinion, therefore, that the ointment must act by some specific power, not merely by excluding the air.

The degree of preservative influence exerted by vaccination, and the propriety of revaccination, continue still to be debated on the continent, especially at the Academy of Medicine at Paris, where M. Bousquet and M. G. de Claubry take different sides of the question. M. Bousquet* is of opinion that the vaccine virus becomes weakened by its transmission through numerous individuals, and appeals to the fact that the effects produced by the renovated vaccine virus in 1836 were much severer than those which followed vaccination with the old matter. It is, however, a singular fact that the difference between the vesicle produced by the new virus and that produced by the old is not perceptible till the sixth or seventh day, by which time the preservative power of vaccination has been exerted, as is shown by the circumstance that a second vaccination after that day does not produce any effect. Still the increased frequency of variolous and varioloid affections of late years, and the fact that the greater number of persons in whom smallpox occurs after vaccination are adults, induce M. Bousquet to believe that a weakening of the vaccine virus does result from its repeated transmission through different individuals, and that the preservative power of vaccination does not extend beyond a certain term of years, though its modifying power continues during the whole of life. He inclines, therefore, to the adoption of revaccination. M. G. de Claubry,† on the other hand, insists on the absence of any ratio between the severity of the local effects of vaccination and its preservative power, and regards the symptoms which have followed vaccination practised direct from the cow merely as phenomena attending the naturalization of cowpox in the human subject. When this has once been accomplished, the transmission of the virus through hundreds of individuals in no way impairs its virtues or modifies the character of the eruption which it occasions. In proof of this he mentions that in some places the same virus has been employed for twenty-five, thirty, or even forty years; and yet the character of the vaccine vesicle has continued unchanged during the whole time. No argument for revaccination can be drawn from the occurrence of secondary smallpox, since it occurs even in persons who have had variola once in the natural way; and M. de Claubry states, though on rather slender grounds, that this takes place in one of every sixty-three persons who have been attacked by the natural smallpox. He asserts too that varioloid eruptions were met with in the early days of vaccination as frequently as now, though their variolous nature was not then apprehended; and denies that such eruptions are by any means confined to adults. He finally endeavours to weaken the positive evidence in favour of revaccination, by showing that varicella, varioloid eruptions, and variola have occurred in the Prussian army among persons who had been revaccinated.

The results of recently performed revaccination in the Prussian army, and in a district of Silesia, are contained in the documents referred to below.‡

Dr. Kahlert, of Prague, has made the experiment of passing vaccine lymph from the human subject through the cow, with a view to increase its activity. This experiment, which is the same as M. Bousquet and Mr. Ceely performed, was quite successful. The retro-vaccine lymph thus obtained produced very characteristic vaccine vesicles in some children who were inoculated with it, but without causing any peculiarly severe constitutional disturbance.§

*Remittent or typhoid fever.* MM. Rilliet and Barthez's work|| contains an elaborate essay on this disease, founded on the analysis of 111 observations In 29 cases the disease proved fatal; and the appearances found on a post-mortem examination furnish the writers with grounds for their opinion that it is identical with typhoid fever in the adult. The lesions of Peyer's glands, of the solitary glands, and of the mesenteric glands are precisely the same as are met with in the adult, except that the ulcerations are usually smaller, shallower, and less numerous. Ulceration, too, is not the constant result of inflammation of Peyer's glands in childhood, since resolution

* Bulletin de l'Acad. Royale de Médecine, Oct. 15, 1842, and Sept. 30 and Oct. 15, 1843.
† Ib., Sept. 30, and Oct. and Nov. 1843.
‡ Med. Zeitung, Jan. 18, and April 5, 1843.
§ Oest. med. Jahrbücher, June, July, and Aug. 1843.
|| Lib. cit., tome ii, p, 350.

sometimes takes place, and when it does not the advance of the ulceration is slower and its cicatrization more rapid than in the adult. Typhoid fever occurs most frequently from nine to fourteen years of age; it is less frequent from five to eight; and very unusual in the earlier years of childhood. MM. Rilliet and Barthez describe three forms of it, differing from each other in intensity; the first being very slight, the third very severe, the second intermediate in intensity between the other two. The symptoms of the first form, which they met with in 47 cases, are much the same as those which characterize what is ordinarily called infantile remittent fever in this country. Both this and the other forms, however, present some peculiarities in their symptoms, and run an unfavorable course more frequently than is the case with the remittent fever with which English writers are familiar; a difference probably owing in part to the unfavorable circumstances in which patients are placed in the Hôpital des Enfans at Paris. The writers enlarge on the diagnosis between remittent fever, meningitis, pneumonia, and enteritis; but conclude that when enteritis assumes a typhoid character its distinction from typhoid fever is almost impossible; though the appearances found after death are very different. The remarks on the treatment of the disease do not present anything new, except the recommendation of the sulphate of quinine in the ataxic and adynamic forms: but the whole essay will well repay a careful perusal.

DYSCRASIÆ.

*Gangrenes.* M. Becquerel* notices the connexion that often exists between pseudo-membranous and gangrenous affections in childhood, and illustrates his remarks by the description of an epidemic which prevailed in the year 1841, at the Hôpital des Enfans. The two forms of disease presented close relations to each other; both causing to a great extent the same general symptoms, and one succeeding to or complicating the other. The epidemic croup of 1840, did not altogether cease at the close of that year, and again became epidemic in the ensuing year, and at the same time its complication with gangrenous affections began to occur. M. Becquerel observed seventeen cases of gangrenous angina, twenty cases of croup, and eighteen in which blistered surfaces became gangrenous.

Gangrene of the pharynx was usually preceded by pseudo-membranous angina. Sometimes it coincided with gangrene of blistered surfaces, or of other parts, and in these cases was only a repetition of the morbid process which existed elsewhere. In most cases of gangrene of the pharynx there existed a preternaturally liquid state of the blood, and the symptoms that existed during its progress were such as betokened a generally adynamic condition. It was frequently complicated with pulmonary apoplexy, occasionally with hemorrhage from the bowels. Pneumonia was an unusual complication. It was treated, unavailingly by local cauterization, and by general tonics.

Croup occurred in twenty cases. It often succeeded to a pseudo-membranous angina, or came on as an idiopathic affection. It seldom followed the eruptive fevers. In addition to the ordinary symptoms of croup, phenomena indicative of disturbance of the nervous system often came on towards its close, and pneumonia was frequent. All the cases terminated fatally; tracheotomy having been practised nine times without success.

Gangrene of blistered surfaces took place in the course of very various diseases; but mostly of such as presented something of an adynamic character. It often came on in the course of measles. False membranes usually formed over the blistered surface; which next became gangrenous, but the patients died under symptoms of great exhaustion before the eschar became detached. Local treatment and general tonics invariably failed of success.

MM. Rilliet and Barthez† give a full description of the different forms of gangrene which occur in childhood, and regard them all, not as mere local maladies which subsequently react on the constitution, but as the result of a generally diseased state of the whole organism. M. Boudet‡ in the course of some remarks on gangrene of the lung in childhood, observes that spontaneous gangrenes in children, appear invariably to develop themselves under the influence of causes acting on the entire economy. He conceives the proximate cause of all these affections to be an altered state of the

* Gazette Médicale, Oct. 28 and Nov. 4, 11, 18, 1843. † Lib. cit., tome ii, p. 98.

‡ Archives Gén. de Médecine, Août et Sept. 1843.

blood, such as succeeds scurvy, measles, and scarlatina, and which is characterized during life by hemorrhage, purpurous spots, ecchymoses, &c. The lesion of the blood, consists in his opinion in a diminution of its fibrin, and an excess of alkali. This supposition, however, rests on but a slender basis; partly on the statement of MM. Andral and Gavarret, that the fibrin of the blood is diminished, and its fluidity increased in the exanthemata; partly on the result of his own investigations. He examined the blood carefully four times: twice it was entirely fluid in the heart and vessels, twice it was diffluent, and scarcely coagulated at all; and in the remaining cases, ten in number, it was much less firmly coagulated than natural.

M. Huguier* relates a case of that rare occurrence, *spontaneous gangrene of the surface* in a child aged 7½ years. The previous history of the patient was unknown, beyond his own statement, that two months before he had had a very violent cold. When received into the Charité, the tips of all the fingers of the left hand were black, and those of the right hand had begun to show a darkish colour. All the toes of the left foot were quite black, the middle toe had dropped off, and the tips of the toes of the right foot were also black. There was neither pain nor redness in the course of the arteries, nor were they less yielding and elastic than natural. The child was bled twice, and took opium during his stay in the hospital; the duration of which, as well as many other points of importance in the case, are not stated. He left the hospital well, having lost all the toes of both feet, the fingers of the left hand, and the whole of the right hand, as also the skin at the tip of the nose, at the prominence of the cheek-bones, the chin, and the edges of the ears.

*Tubercle and scrofula.* MM. Rilliet and Barthez† contend earnestly for the identity of the tuberculous and scrofulous diseases; and they assert, that having examined the bodies of a large number of scrofulous children at the Hôpital St. Louis, they met with no instance in which tubercular deposits did not exist in some part or other. They regard many affections, usually termed scrofulous, such as ophthalmia, caries, eczema, &c., as being of a secondary nature, accidentally complicating the original scrofulous, or tuberculous habit, but not essentially scrofulous in their nature. They propose to banish the name, scrofula, from medical nomenclature, as being vague, and likely to mislead, and to substitute for it the term tuberculization. M. Bredow,‡ of St. Petersburgh, takes a similar view of the identity of the two diseases; he proposes, however, to retain the word scrofula, to designate tuberculous disorganization of the lymphatic glands, and to employ the name, tubercle and tuberculous disease for the same affection existing in other organs. The interesting researches of MM. Rilliet and Barthez into the anatomy of tubercle, belong properly to the domain of pathological anatomy. They have directed their attention to some other points, however, which may with propriety be mentioned here. The extreme frequency of tuberculous disease in childhood appears from the fact, that it existed in a more or less advanced state, in some part or other, in 314 out of 525 children, of whom they made a post-mortem examination. With reference to the influence of sex and age on its production, they find that it is most frequent from 6 to 10½ years of age, then from 11 to 15, next from 2 to 5, and lastly from 1 to 2½; and they support this statement as well as their other assertions by statistical data. The female sex is, on the whole, more liable to it than the male, though this liability is not the same at all ages, for from 1 to 2½ years of age more cases are met with in the male; from 3 to 5 there is a slight excess among females, and from 6 to 10 the two sexes seem equally liable to the disease, but from 11 to 15, that is to say, at the time when puberty approaches, female children suffer much more from it than males. It appears, too, that tuberculous deposit is most frequently met with only in a slight degree from 3 years of age to 5½; that a moderately abundant deposit is oftenest found from 1 to 2, and next in frequency from 3 to 5; and that very abundant deposit is much oftener discovered from 6 to 15, than from 1 to 5 years of age. Children under 1 year old, did not come under their notice.

They give an interesting table of the comparative frequency of tuberculous deposit in different organs in 314 children between the ages 1 and 15:

* Clinique des Hôpitaux des Enfans, Dec. 1842.

† Lib. cit., tome iii, pp. 1-163.

‡ Ueber die Scrofelsucht, 8vo; Berlin, 1843.

| Tubercle was present in the | | Tubercle was present in the | |
|---|---|---|---|
| Lungs . . . | in 265 cases. | Liver . . . | in 71 cases. |
| Bronchial Glands . | „ 219 „ | Large Intestines . | „ 60 „ |
| Mesenteric Glands . | „ 144 „ | Membranes of the Brain | „ 52 „ |
| Small Intestines . | „ 134 „ | Kidneys . . | „ 49 „ |
| Pleura . . . | „ 109 „ | Brain . . . | „ 37 „ |
| Spleen . . | „ 107 „ | Stomach . . | „ 21 „ |
| Peritoneum . . | „ 86 „ | Pericardium, Heart . | „ 10 „ |

The work of M. Bredow* is an extremely well-executed compendium on the subject of scrofula, and contains many valuable practical suggestions derived from his extensive experience as physician to the Imperial Manufactory of Alexandrowsk, near St. Petersburgh.

*Rickets.* Dr. Elsässer† opposes the commonly received opinion, that this disease does not occur during infantile life; and describes a peculiar affection of the skull in early infancy, which he believes to be the result of rickets. It consists in softening of the cranial bones, attended with increase of vascularity and alteration of their texture, which becomes spongy, rough, and porous, owing to a diminution of their earthy constituents, and disintegration of their tissue. Under the microscope the canaliculi of the bones are seen to be dilated, and communicate freely with each other; —conditions that have been observed to follow from the action of rickets on other bones. To this affection he gives the name of *craniotabes*, or *the soft occiput*, from that yielding of the bones under pressure which constitutes one of its most striking symptoms. A great part of infantile life being spent in the recumbent posture, the softened and yielding occiput has to support the pressure of the contents of the cranium for many hours daily. This circumstance would of itself produce some unpleasant effects, which are aggravated by the tendency of the pulsation of the brain, within the cranium, and the pressure of the pillow on which the infant reposes, without the cranium, to attenuate the softened bone at those places where the convolutions of the brain are most prominent. Sometimes the bony matter is completely absorbed in various places, the holes in the skull being closed merely by the dura mater and pericardium. This condition is not the result of originally defective ossification, for it is not met with in the fœtus; and, moreover, the situation of the openings in the skull does not correspond with those which would be produced by any suspension of the process of ossification. It is not found either immediately after birth, but begins about the third month, or somewhat later; and the circumstance that the attenuated and perforated spots are found only at the occiput or the occipital end of the parietal bones, though the whole cranium is softer than natural, proves that it is the result of pressure exerted in the way already described. It is but natural that, the protecting case of the brain being thus weakened and thinned, the organ itself should suffer from various external agents. Accordingly, cerebral irritation and congestion are produced, and show themselves in convulsive seizures of various kinds, and frequently in attacks closely resembling laryngismus stridulus. So far, too, is the affection from being one of slight moment, that half of those who are affected by it die; while its occurrence is so frequent that Dr. Elsässer has met with it forty times, in the course of five years' practice, in a small country town. The appropriate treatment consists in the employment of tonics, among which iron bears the chief place; and in the adoption of various precautions to prevent or, at least, to diminish pressure on the occiput. [Notwithstanding Dr. Elsässer's assertion to the contrary, a suspicion may be entertained that he has been misled as to the frequency of this affection; or that endemic causes or some local peculiarities have tended to render it very prevalent in the town where he practises. For the last six months, since his work came under the notice of the writer of this Report, he has carefully examined the heads of infants who came under his care at the Infirmary for Children, and has very rarely found any such yielding of the bones as Dr. Elsässer frequently met with, except just in the neighbourhood of the anterior fontanelle. Laryngismus stridulus, too, has not come under the writer's notice more than two or three times a year, during the past five years, notwithstanding the large number of children who are brought to the Institution.]

* Ueber die Scrofelsucht, 8vo; Berlin, 1843.

† Der weiche Hinterkopf, etc, 8vo; Stuttgart und Tübingen, 1843. The present Number of this Review contains a more extended analysis of Dr. Elsässer's observations.

